# Intrathecal trastuzumab versus alternate routes of delivery for HER2-targeted therapies in patients with HER2+ breast cancer leptomeningeal metastases

**DOI:** 10.1016/j.breast.2023.04.008

**Published:** 2023-05-01

**Authors:** Anna-Maria Lazaratos, Sarah M. Maritan, Andrea Quaiattini, Amelie Darlix, Ivica Ratosa, Emanuela Ferraro, Gaia Griguolo, Valentina Guarneri, Alessia Pellerino, Silvia Hofer, William Jacot, Hans-Joachim Stemmler, Marcel P.H. van den Broek, Nika Dobnikar, Francois Panet, Zubin Lahijanian, Aki Morikawa, Andrew D. Seidman, Riccardo Soffietti, Lawrence Panasci, Kevin Petrecca, April A.N. Rose, Nathaniel Bouganim, Matthew Dankner

**Affiliations:** a Rosalind and Morris Goodman Cancer Institute, Montreal, Quebec, Canada; b Faculty of Medicine, Université de Montréal, Montreal, Quebec, Canada; c Faculty of Medicine, Division of Experimental Medicine, McGill University, Montreal, Quebec, Canada; d Schulich Library of Physical Sciences, Life Sciences, and Engineering, McGill University, Montreal, Quebec, Canada; e Department of Medical Oncology, Institut régional du Cancer de Montpellier, University of Montpellier, Montpellier, France; f Institut de Génomique Fonctionnelle, INSERM, CNRS, University of Montpellier, Montpellier, France; g Division of Radiotherapy, Institute of Oncology Ljubljana, Ljubljana, Slovenia; h Faculty of Medicine, University of Ljubljana, Ljubljana, Slovenia; i Breast Cancer Service, Department of Medicine, Memorial Sloan Kettering Cancer Center, NewYork, USA; j Division of Oncology 2, Istituto Oncologico Veneto IRCCS, Padova, Italy; k Department of Surgery, Oncology and Gastroenterology, University of Padova, Padova, Italy; l Division of Neuro-Oncology, Department of Neuroscience, University and City of Health and Science Hospital, Turin, Italy; m Department of Neurology, University Hospital Zurich, Switzerland; n Medical Dept. III, Ludwig Maximilians University Munich, Munchen, Germany; o Clinical Pharmacy, Sint Antonius Hospital, Nieuwegein, the Netherlands; p Gerald Bronfman Department of Oncology, Faculty of Medicine and Health Sciences, McGill University, Montreal, Quebec, Canada; q Department of Diagnostic Radiology, McGill University, Montreal, Quebec, Canada; r Montreal Neurological Institute, McGill University, Montreal, Quebec, Canada; s Lady Davis Institute, Segal Cancer Centre, Jewish General Hospital, McGill University, Montreal, Quebec, Canada; t McGill University Health Centre, McGill University, Montreal, Quebec, Canada

**Keywords:** Breast cancer, Leptomeningeal, Intrathecal, Trastuzumab, Deruxtecan

## Abstract

**Background:**

Patients with HER2+ breast cancer (BC) frequently develop leptomeningeal metastases (LM). While HER2-targeted therapies have demonstrated efficacy in the neoadjuvant, adjuvant, and metastatic settings, including for parenchymal brain metastases, their efficacy for patients with LM has not been studied in a randomized controlled trial. However, several single-armed prospective studies, case series and case reports have studied oral, intravenous, or intrathecally administered HER2-targeted therapy regimens for patients with HER2+ BC LM.

**Methods:**

We conducted a systematic review and meta-analysis of individual patient data to evaluate the efficacy of HER2-targeted therapies in HER2+ BC LM in accordance with PRISMA guidelines. Targeted therapies evaluated were trastuzumab (intrathecal or intravenous), pertuzumab, lapatinib, neratinib, tucatinib, trastuzumab-emtansine and trastuzumab-deruxtecan. The primary endpoint was overall survival (OS), with CNS-specific progression-free survival (PFS) as a secondary endpoint.

**Results:**

7780 abstracts were screened, identifying 45 publications with 208 patients, corresponding to 275 lines of HER2-targeted therapy for BC LM which met inclusion criteria. In univariable and multivariable analyses, we observed no significant difference in OS and CNS-specific PFS between intrathecal trastuzumab compared to oral or intravenous administration of HER2-targeted therapy. Anti-HER2 monoclonal antibody-based regimens did not demonstrate superiority over HER2 tyrosine kinase inhibitors. In a cohort of 15 patients, treatment with trastuzumab-deruxtecan was associated with prolonged OS compared to other HER2-targeted therapies and compared to trastuzumab-emtansine.

**Conclusions:**

The results of this meta-analysis, comprising the limited data available, suggest that intrathecal administration of HER2-targeted therapy for patients with HER2+ BC LM confers no additional benefit over oral and/or IV treatment regimens. Although the number of patients receiving trastuzumab deruxtecan in this cohort is small, this novel agent offers promise for this patient population and requires further investigation in prospective studies.

## Highlights

• Meta-analysis of HER2-targeted therapies for breast cancer leptomeningeal metastases.
• Intrathecal trastuzumab is not superior to intravenous or oral anti-HER2 therapies.
• Trastuzumab deruxtecan demonstrates impressive efficacy in this setting.

## Graphical abstract

**Figure:**
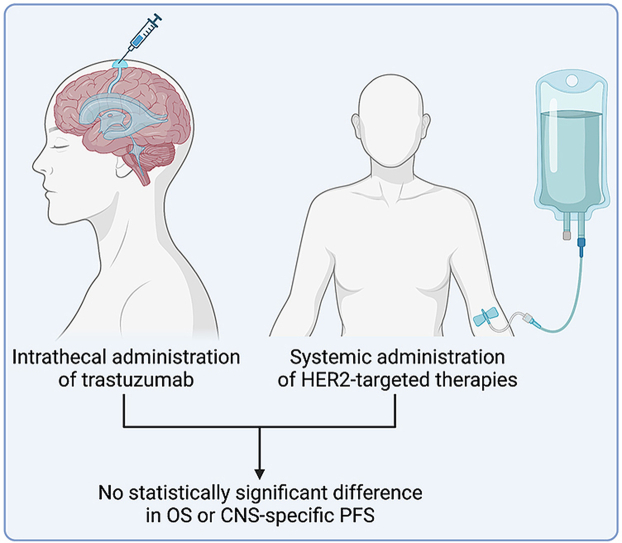

## 1 Introduction

Leptomeningeal metastasis (LM), also known as neoplastic meningitis or leptomeningeal carcinomatosis, is a debilitating condition associated with advanced breast cancer (BC) [1]. LM is defined by cancer cells reaching and proliferating in the subarachnoid space that surrounds the brain and spinal cord [1]. The development of LM portends a dismal prognosis for BC patients, with median overall survival (mOS) measured in weeks to months [2].

When HER2 amplification or overexpression is present (HER2+), monoclonal antibody, antibody-drug conjugate, and small-molecule targeted therapies represent important components of the treatment armamentarium for BC LM. These include trastuzumab, pertuzumab, lapatinib, neratinib, tucatinib, trastuzumab-emtansine (T-DM1), and trastuzumab-deruxtecan (T-DXd). LM occurs in 6–12% of patients with HER2+ BC and in up to 24% of patients with HER2+ BC parenchymal brain metastases [3,4].

For patients with HER2+ BC LM, no randomized controlled trials have been performed comparing HER2-targeted regimens. However, seven single-armed prospective studies assessing HER2-targeted therapies for BC LM have been published to date [[5], [6], [7], [8], [9], [10], [11]]. Three of these trials studied the safety and activity of intrathecal (IT) trastuzumab [[5], [6], [7]]. This has led to uptake of this treatment approach in many centers globally despite its modest but clear morbidity [7]. It remains uncertain whether IT administration of trastuzumab confers any tangible benefit for patients with HER2+ BC LM compared to other approaches with respect to meaningful clinical endpoints, such as quality-of-life, progression-free survival (PFS), and overall survival (OS).

To evaluate the efficacy of HER2-targeted therapy in the management of BC LM, we performed a systematic review and meta-analysis of all published data on clinical outcomes in patients with HER2+ BC LM treated with HER2-targeted therapies. This has allowed us to make the first comparisons between IT versus intravenous (10.13039/501100000026IV) or oral HER2-targeted therapies for BC 10.13039/100006186LM and present the first evidence supporting the efficacy of T-DXd compared to alternative strategies for this patient population.

## 2 **Methods**

**Search Strategy:** A literature search was conducted of studies published from January 1964 to December 2021 in the following databases: Medline ALL (Medline and Medline Epub Ahead of print and In-Process & Other Non-Indexed Citations), Embase, Cochrane Central Register of Controlled Trials, Scopus, and Web of Science Core Collection. The detailed search strategy is presented in Appendix 1. Published conference abstracts were included. Additional publications and/or data identified by the authors outside of the search were added to the systematic review when applicable. The study protocol was prospectively uploaded to PROSPERO (ID: CRD42021292539) and followed the Preferred Reporting Items for Systematic Reviews and Meta-Analyses guidelines (PRISMA) [12,13].

All abstracts were screened by two independent reviewers (reviewers included authors AML, SMM and MD) using the Rayyan software (www.rayyan.ai). Conflicts were resolved with internal discussion between the three reviewers (AML, SMM, MD). For any publications for which a consensus could not be reached (N = 5 abstracts), a fourth reviewer (NB) made the determination to include or exclude. Of the 32 articles extracted by the authors of this manuscript without obtaining data from authors of the source manuscript, 50% (16) articles were independently extracted by two reviewers (AML and MD), revealing 100% concordance between both reviewers. The remaining 16 articles were extracted by a single reviewer (AML).

After data was extracted from all included publications, missing data was identified and requested from the original authors of each publication with two separate email prompts >7 days apart. Clinical information from 93 patients, corresponding to 117 lines of therapy, were extracted by the authors of this manuscript from the source publications, while data for 115 patients, corresponding to 158 lines of therapy, were obtained through communication with the authors of the corresponding publication (Appendix 2). We unsuccessfully attempted to obtain data from an additional 16 retrospective and 7 prospective studies, corresponding to 343 patients (Appendix 3). Together, we captured approximately 38% of HER2+ BC LM patients treated with HER2-targeted therapies described in the literature. However, this is likely an underestimate of the true percentage captured because the number of patients described in many of the studies we unsuccessfully attempted to obtain data from describe general patient populations that would require further refinement based on our inclusion and exclusion criteria.

**Inclusion and exclusion criteria:** Inclusion criteria were as follows: adult HER2+ BC patients (aged 18 years or older), defined by 3+ immunohistochemistry (IHC) staining or 2+ IHC with fluorescence *in situ* hybridization (FISH) demonstrating HER2 amplification, with a diagnosis of LM defined on magnetic resonance imaging (MRI) or with positive cerebrospinal fluid (CSF) cytology and receiving a HER2-targeted therapy for the treatment of LM. HER2-targeted therapies evaluated included trastuzumab (IT or IV), pertuzumab, lapatinib, tucatinib, neratinib, T-DM1 and T-DXd (Supplemental Table S1). Hormone receptor positivity was defined by the authors of each individual study incorporated in the meta-analysis. There was one male patient included in our study. Radiotherapy (RT) employed for the treatment of LM included stereotactic radiosurgery (SRS), whole-brain RT (WBRT) and spinal RT.

**Primary and secondary outcomes:** The primary outcome was OS, which was calculated from the start of HER2-targeted therapy for LM. The secondary outcome was CNS-specific PFS, which was calculated based on central nervous system (CNS)-specific progression, or death. Progression was defined by the primary paper's author's assessment via MRI or CSF analysis, or death of the patient. Statistical analyses in Table 2, Table 3 were performed with available individual patient data.

**Table 1:** Individual patient characteristics.

**Variable**	**Entire Cohort No. (%)**	**No IT cohort No. (%)**	**IT cohort No. (%)**	**P (Fisher's exact)**	**Pearson's χ2**
**Patient-lines of therapy**	**275**	**183 (66.55)**	**92 (33.45)**		
**Study characteristics**					
**Geographic location**					
North America	85 (30.90)	43 (23.5)	42 (45.65)	**<0.001**	**20.715, P < 0.001**
Europe	183 (66.55)	138 (75.41)	45 (48.91)	**<0.001**	
Asia	7 (2.55)	2 (1.09)	5 (5.44)	**0.044**	
**Year of study**					
<2018	73 (26.55)	38 (20.77)	35 (38.04)	**0.004**	
≥2018	202 (73.45)	145 (79.23)	57 (61.96)		
**Sample size**					
<5	71 (25.82)	33 (18.03)	38 (41.3)	**<0.001**	
≥5	204 (74.18)	150 (81.97)	54 (58.7)		
**Risk of bias**					
≤3	33 (12)	19 (10.38)	14 (15.22)	0.245	
4, 5	242 (88)	164 (89.62)	78 (84.78)		
**Study type**					
Retrospective	256 (93.09)	180 (98.36)	76 (82.61)	**<0.001**	
Prospective	19 (6.91)	3 (1.64)	16 (17.39)		
**Patient characteristics**					
**Age, years**					
<60	173 (62.91)	103 (56.28)	70 (76.09)	**0.018**	
≥60	84 (30.55)	63 (34.43)	21 (22.83)		
Unknown	18 (6.54)	17 (9.29)	1 (1.08)		
**Hormone receptor status**					
Hormone receptor negative	23 (8.36)	12 (6.56)	11 (11.96)	**0.013**	
Hormone receptor positive	62 (22.55)	50 (27.32)	12 (13.04)		
Unknown	190 (69.09)	121 (66.12)	69 (75)		
**Lines of therapy in metastatic setting**					
0–1	52 (18.91)	36 (19.67)	16 (17.39)	**0.012**	
≥2	163 (59.27)	140 (76.5)	23 (25)		
Unknown	60 (21.82)	7 (3.83)	53 (57.61)		
**Prior anti-HER2 targeted therapy**					
No	11 (4)	6 (3.28)	5 (5.44)	0.3	
Yes	151 (54.91)	109 (59.56)	42 (45.65)		
Unknown	113 (41.09)	68 (37.16)	45 (48.91)		
**Concurrent extracranial metastases**					
No	44 (16)	25 (13.66)	19 (20.65)	**0.045**	
Yes	204 (74.18)	149 (81.42)	55 (59.78)		
Unknown	27 (9.82)	9 (4.92)	18 (19.57)		
**Concurrent intracranial metastases**					
No	65 (23.64)	52 (28.41)	13 (14.13)	0.187	
Yes	163 (59.27)	116 (63.39)	47 (51.09)		
Unknown	47 (17.09)	15 (8.2)	32 (34.78)		
**Location of LM**					
Brain	143 (52)	121 (66.12)	22 (23.91)	**0.011**	**7.505, P = 0.023**
Spinal cord	10 (3.64)	6 (3.28)	4 (4.35)	0.117	
Both	46 (16.73)	32 (17.49)	14 (15.22)	0.059	
Unknown	76 (27.63)	24 (13.11)	52 (56.52)		
**ECOG status**					
0–1	89 (32.37)	85 (46.44)	4 (4.35)	0.471	
≥2	53 (19.27)	49 (26.78)	4 (4.35)		
Unknown	133 (48.36)	49 (26.78)	84 (91.3)		
**Diagnostic characteristics**					
**Method of LM diagnosis**					
MRI	80 (29.09)	72 (39.34)	8 (8.7)	**0.007**	**28.872, P < 0.001**
CSF	41 (14.91)	35 (19.13)	6 (6.52)	0.511	
Both	71 (25.82)	44 (24.04)	27 (29.35)	**<0.001**	
Other	15 (5.45)	15 (8.2)	0 (0)	**0.046**	
Unknown	68 (24.73)	17 (9.29)	51 (55.43)		
**Time from primary cancer diagnosis to LM diagnosis, months**					
<48	38 (13.82)	22 (12.02)	16 (17.39)	**0.045**	
≥48	44 (16)	15 (8.2)	29 (31.52)		
Unknown	193 (70.18)	146 (79.78)	47 (51.09)		
**LM treatment characteristics**					
**Type of therapy**					
Monoclonal antibody	212 (77.09)	129 (70.49)	83 (90.22)	**<0.001**	**23.532, P < 0.001**
Small molecule inhibitor	40 (14.55)	40 (21.86)	0 (0)	**<0.001**	
Both	23 (8.36)	14 (7.65)	9 (9.78)	0.645	
**Type of monoclonal antibody**					
Non-ADC monoclonal antibody	183 (66.55)	93 (50.82)	90 (97.83)	**<0.001**	
ADC	52 (18.91)	50 (27.32)	2 (2.17)		
**Regimens including each of the following therapies**					
**Trastuzumab-based anti-HER2 treatments**					
Trastuzumab	185	93	92	**<0.001**	
T-DXd	15	14	1^a^	**0.024**	
T-DM1	37	36	1^a^	**<0.001**	
Pertuzumab + trastuzumab	20	20	0	**<0.001**	
**Non-trastuzumab-based anti-HER2 treatments**					
Lapatinib	46	38	8	**0.011**	
Neratinib	11	10	1	0.106	
Tucatinib	6	6	0	0.183	
**ADC for LM (T-DM1 or T-DXd)**					
No	223 (81.09)	133 (72.68)	90 (97.83)	**<0.001**	
Yes	52 (18.91)	50 (27.32)	2 (2.17)		
**ADC type (T-DXd vs T-DM1) for LM**					
T-DM1	37 (13.45)	36 (19.67)	1 (1.09)	0.498	
T-DXd	15 (5.45)	14 (7.65)	1 (1.09)		
**IT trastuzumab versus non-ADC monoclonal antibody**					
IV non-ADC monoclonal antibody	93 (33.82)	93 (50.82)	0 (0)	**<0.001**	
IT trastuzumab	92 (33.45)	0 (0)	92 (100)		
**IT trastuzumab versus ADC**					
IV ADC	52 (18.91)	50 (27.32)	2 (2.17)	**<0.001**	
IT trastuzumab	90 (32.73)	0 (0)	90 (97.83)		
**Trastuzumab-based regimens**					
No	40 (14.55)	40 (21.86)	0 (0)	**<0.001**	
Yes	235 (85.45)	143 (78.14)	92 (100)		
**Chemotherapy simultaneously with anti-HER2 targeted therapy**					
No	84 (30.55)	29 (15.85)	55 (59.78)	**<0.001**	
Yes	191 (69.45)	154 (84.15)	37 (40.22)		
**Route of administration for chemotherapy concurrently to HER2 targeted therapy**					
IT only	26 (9.45)	6 (3.28)	20 (21.74)	**<0.001**	**63.837, P < 0.001**
IV or oral only	138 (50.18)	124 (67.76)	14 (15.22)	**<0.001**	
IT and IV/oral	27 (9.82)	24 (13.11)	3 (3.26)	0.302	
**Radiotherapy for LM**					
No	124 (45.09)	74 (40.44)	50 (54.35)	**0.04**	
Yes	150 (54.55)	108 (59.02)	42 (45.65)		
Unknown	1 (0.36)	1 (0.54)	0 (0)		
**Type of radiotherapy for LM**					
Stereotactic radiosurgery	2 (0.73)	1 (0.55)	1 (1.09)	0.483	**77.103, P < 0.001**
Whole-brain radiotherapy	52 (18.91)	15 (8.2)	37 (40.22)	**<0.001**	
Spinal radiotherapy	10 (3.64)	9 (4.92)	1 (1.09)	0.284	
Whole-brain radiotherapy and spinal radiotherapy	11 (4)	9 (4.92)	2 (2.17)	0.729	
Unknown	75 (27.27)	74 (40.44)	1 (1.09)		

**NOTE.** Patient characteristics were compared between those who received intrathecal trastuzumab and those who did not, with Fisher’s Exact test and Pearson’s X^2^. Bold values indicate P < 0.05.

**Abbreviations:** LM, leptomeningeal metastasis; HER2, human epidermal receptor 2; CSF, cerebrospinal fluid; MRI, magnetic resonance imaging; ECOG, Eastern Cooperative Oncology Group; ADC, antibody-drug conjugate T-DXd, trastuzumab-deruxtecan; T-DM1, trastuzumab-emtansine; IV, intravenous; IT, intrathecal.

a T-DM1 and T-DXd were administered intravenously in the context of the patient receiving intrathecal trastuzumab simultaneously.

**Table 2:** Overall survival rates associated with clinical variables.

**Characteristics**	**Patient lines of therapy**	**Median OS (months)**	**Univariable Hazard Ratio**	**Univariable 95% CI**	**Univariable P value**	**Adjusted Hazard Ratio**	**Multivariable 95% CI**	**Adjusted P Value**
Entire Cohort	275	14.29						
**Study characteristics**								
**Geographic location**								
North America	85	14	0.736	0.493–1.098	0.133			
Europe	183	14.53	1.493	1.009–2.207	**0.045**			
Asia	7	21	0.252	0.034–1.847	0.175			
**Year of study**								
<2018	73	19	1.242	0.844–1.827	0.271			
≥2018	202	13.44						
**Sample size**								
<5	71	25	1.783	1.217–2.610	**0.003**			
≥5	204	12.89						
**Risk of bias**								
≤3	33	20	1.105	0.661–1.848	0.703			
4, 5	242	14.23						
**Study type**								
Retrospective	256	14.82	1.452	0.773–2.729	0.247			
Prospective	19	11.78						
**Patient characteristics**								
**Age, years**								
<60	173	19	1.676	1.227–2.289	**0.001**			
≥60	84	12						
**Hormone receptor status**								
Hormone receptor negative	23	46	3.293	1.362–7.960	**0.008**			
Hormone receptor positive	62	20						
**Lines of therapy in metastatic setting**								
0–1	52	28.73	1.837	1.196–2.821	**0.005**			
2 or more	163	14						
**Previous HER2-targeted therapy**								
No	11	25.26	2.453	0.871–6.911	0.09			
Yes	151	19						
**Concurrent extracranial metastasis**								
No	44	25.7	1.442	0.949–2.190	0.086			
Yes	204	14						
**Concurrent intracranial metastasis**								
No	65	15	1.095	0.763–1.570	0.623			
Yes	163	14.59						
**Location of LM**								
Brain	143	14.08	1.217	0.807–1.836	0.348			
Spinal cord	10	12.53	1.43	0.658–3.110	0.367			
Both	46	25.26	0.72	0.464–1.116	0.142			
**ECOG status**								
0, 1	89	14.29	2.186	1.478–3.232	**<0.001**	2.16	1.458–3.120	**<0.001**
2, 3, 4	53	8.53						
**Diagnostic characteristics**								
**Method of LM diagnosis**								
MRI	80	14.08	0.791	0.531–1.178	0.248			
CSF	41	19.07	1.096	0.688–1.745	0.699			
Both	71	17	0.939	0.617–1.427	0.767			
Other	15	12.58	1.61	0.905–2.864	0.105			
**Time from primary diagnosis to LM diagnosis, months**								
<48 months	38	15	0.889	0.435–1.816	0.746			
≥48 months	44	21						
**LM treatments**								
**Type of therapy**								
Monoclonal antibody	212	14.08	1.14	0.787–1.653	0.488			
Small molecule inhibitor	40	14.23	1.133	0.755–1.699	0.547			
Both	23	20	0.587	0.319–1.079	0.087			
**Type of monoclonal antibody**								
Non-ADC monoclonal Ab	183	14.2	0.835	0.541–1.291	0.418			
ADC	52	21						
**Regimens including each of the following therapies**								
**Trastuzumab-based anti-HER2 treatments**								
Trastuzumab	185	14.29	1.036	0.748–1.436	0.831			
T-DXd	15	N/A	0.253	0.062–1.038	0.056			
T-DM1	37	14.23	1.049	0.672–1.636	0.834			
Pertuzumab + trastuzumab	20	17.94	0.883	0.504–1.547	0.664			
**Non-trastuzumab-based anti-HER2 treatments**								
Lapatinib	46	21	0.742	0.491–1.222	0.158			
Neratinib	11	10	2.115	1.009–4.443	**0.047**			
Tucatinib	6	14	0.699	0.169–2.897	0.622			
**ADC for LM (T-DM1 or T-DXd)**								
No	233	14.2	0.826	0.538–1.268	0.383			
Yes	52	21						
**ADC type (T-DXd vs T-DM1) for LM**								
T-DM1	37	14.23	0.224	0.053–0.958	**0.044**			
T-DXd	15	N/A						
**IT trastuzumab**								
No	183	14.23	0.919	0.633–1.334	0.657	1.458	0.687–3.092	0.325
Yes	92	14.53						
**IT trastuzumab versus non-ADC mAb**								
IV non-ADC mAb	93	14.2	0.814	0.566–1.171	0.268			
IT trastuzumab	92	14.53						
**IT trastuzumab versus ADC**								
IV ADC	52	21	1.18	0.674–2.068	0.563			
IT trastuzumab	90	14.53						
**Trastuzumab-based regimens**								
No	40	14.23	0.883	0.589–1.325	0.547			
Yes	235	14.53						
**Chemotherapy for LM simultaneously to HER2 targeted therapy**								
No	84	13.21	0.869	0.626–1.206	0.4			
Yes	191	16.26						
**Route of administration for chemotherapy concurrently to HER2 targeted therapy**								
IT only	26	19.07	0.937	0.509–1.724	0.833			
IV or oral only	138	16.26	0.881	0.553–1.402	0.592			
IT and IV/oral	27	15	1.235	0.716–2.132	0.448			
**Radiotherapy for LM**								
No	124	15	0.817	0.593–1.125	0.216			
Yes	150	14.23						
**Type of radiotherapy for LM**								
Stereotactic radiosurgery	2	46	0.518	0.124–2.160	0.366			
Whole-brain radiotherapy	52	12	0.921	0.584–1.452	0.723			
Spinal radiotherapy	10	20	1.355	0.633–2.902	0.434			
Whole-brain radiotherapy and spinal radiotherapy	11	112.83	0.344	0.106–1.114	0.075			
Unknown	75	14.29	1.414	0.950–2.104	0.088			

**NOTE**. Univariable and multivariable hazard ratios, 95% CIs, and P-values calculated with a multilevel mixed-effects Cox proportional hazards model with article as the random-effects variable. Bold values indicate P < 0.05. **Abbreviations:** CI, confidence interval; mOS, median overall survival; LM, leptomeningeal metastasis; HER2, human epidermal receptor 2; CSF, cerebrospinal fluid; MRI, magnetic resonance imaging; ECOG, Eastern Cooperative Oncology Group; ADC, antibody-drug conjugate T-DXd, trastuzumab-deruxtecan; T-DM1, trastuzumab-emtansine; IV, intravenous; IT, intrathecal.

**Table 3:** CNS-specific progression-free survival rates associated with clinical variables.

**Characteristics**	**Patient lines of therapy**	**Median PFS (months)**	**Univariable Hazard Ratio**	**Univariable 95% CI**	**Univariable P value**	**Adjusted Hazard Ratio**	**Multivariable 95% CI**	**Adjusted P Value**
Entire Cohort	206	6						
**Study characteristics**								
**Geographic location**								
North America	85	5.69	0.943	0.687–1.294	0.716			
Europe	162	7	1.237	0.921–1.662	0.158			
Asia	7	21	0.24	0.060–0.967	**0.045**			
**Year of study**								
<2018	68	7	1.25	0.913–1.710	0.164			
≥2018	186	5.75						
**Sample size**								
<5	71	10	1.668	1.210–2.298	**0.002**			
≥5	183	5.52						
**Risk of bias**								
≤3	32	7	0.949	0.602–1.497	0.822			
4, 5	222	6						
**Study type**								
Retrospective	251	6	0.603	0.188–1.935	0.395			
Prospective	3	20.89						
**Patient characteristics**								
**Age, years**								
<60	158	6	0.929	0.658–1.310	0.673			
≥60	78	7.85						
**Hormone receptor status**								
Hormone receptor negative	21	6	1.794	0.719–4.473	0.21			
Hormone receptor positive	59	7						
**Lines of therapy in metastatic setting**								
0–1	52	8	1.585	1.088–2.309	**0.016**			
2 or more	163	6						
**Previous HER2-targeted therapy**								
No	8	33.83	3.58	1.289–9.940	**0.014**			
Yes	133	7						
**Concurrent extracranial metastasis**								
No	39	7.1	1.34	0.895–2.005	0.155			
Yes	193	6						
**Concurrent intracranial metastasis**								
No	63	7.16	1.145	0.810–1.620	0.444			
Yes	149	7						
**Location of LM**								
Brain	143	7.16	0.85	0.583–1.239	0.398			
Spinal cord	10	5.69	1.002	0.489–2.053	0.997			
Both	46	5	1.184	0.797–1.757	0.403			
**ECOG status**								
0, 1	89	8	1.89	1.310–2.728	**0.001**	1.915	1.323–2.771	**0.001**
2, 3, 4	53	4.27						
**Diagnostic characteristics**								
**Method of LM diagnosis**								
MRI	80	8.54	0.676	0.465–0.983	**0.041**			
CSF	37	6	1.052	0.669–1.655	0.826			
Both	70	6	1.378	0.915–2.075	0.124			
Other	15	5.69	1.333	0.759–2.340	0.317			
**Time from primary diagnosis to LM diagnosis, months**								
<48 months	32	7	0.729	0.396–1.344	0.312			
≥48 months	34	7						
**LM** t**reatments**								
**Type of therapy**								
Monoclonal antibody	193	6	1.17	0.849–1.611	0.338			
Small molecule inhibitor	38	6	0.996	0.678–1.462	0.983			
Both	23	7	0.737	0.458–1.187	0.21			
**Type of monoclonal antibody**								
Non-ADC monoclonal Ab	164	5.75	0.754	0.511–1.113	0.156			
ADC	52	10.51						
**Regimens including each of the following therapies**								
**Trastuzumab-based anti-HER2 treatments**								
Trastuzumab	166	6	1.162	0.859–1.571	0.331			
T-DXd	15	N/A	0.207	0.074–0.582	**0.003**			
T-DM1	37	8.08	1.139	0.768–1.690	0.518			
Pertuzumab + trastuzumab	20	12.16	0.627	0.367–1.073	0.089			
**Non-trastuzumab-based anti-HER2 treatments**								
Lapatinib	44	7.1	0.651	0.449–0.944	**0.024**			
Neratinib	11	4	1.954	0.999–3.824	0.05			
Tucatinib	6	5	1.777	0.775–4.075	0.174			
**ADC for LM (T-DM1 or T-DXd)**								
No	202	6	0.765	0.524–1.117	0.165			
Yes	52	10.51						
**ADC type (T-DXd vs T-DM1) for LM**								
T-DM1	37	8.08	0.265	0.092–0.765	**0.014**			
T-DXd	15	N/A						
**IT trastuzumab**								
No	178	6	0.809	0.595–1.101	0.177	1.252	0.605–2.592	0.544
Yes	76	6.2						
**IT trastuzumab versus non-ADC monoclonal antibody**								
IV non-ADC monoclonal antibody	90	5.52	0.717	0.509–1.008	0.056			
IT trastuzumab	76	6.2						
**IT trastuzumab versus ADC**								
IV ADC	52	10.51	1.416	0.785–2.555	0.247			
IT trastuzumab	74	6.2						
**Trastuzumab-based regimens**								
No	38	6	1.004	0.684–1.474	0.983			
Yes	216	6.2						
**Chemotherapy for LM simultaneously to HER2 targeted therapy**								
No	69	7.16	1.236	0.891–1.715	0.204			
Yes	185	6						
**Route of administration for chemotherapy concurrently to HER2 targeted therapy**								
IT only	25	5.7	1.109	0.695–1.768	0.665			
IV or oral only	138	6	0.919	0.629–1.344	0.665			
IT and IV/oral	22	4.5	1.013	0.615–1.667	0.96			
**Radiotherapy for LM**								
No	103	6.1	1.079	0.810–1.438	0.604			
Yes	150	5.75						
**Type of radiotherapy for LM**								
Stereotactic radiosurgery	2	5.16	1.601	0.392–6.529	0.512			
Whole-brain radiotherapy	52	5.7	0.946	0.646–1.384	0.775			
Spinal radiotherapy	10	5.16	1.441	0.752–2.760	0.27			
Whole-brain radiotherapy and spinal radiotherapy	11	13.49	0.581	0.269–1.254	0.166			
Unknown	75	7.07	1.081	0.756–1.546	0.668			

**NOTE**. Univariable and multivariable hazard ratios, 95% CIs, and P-values calculated with a multilevel mixed-effects Cox proportional hazards model with article as the random-effects variable. Bold values indicate P < 0.05. **Abbreviations:** CI, confidence interval; mPFS, CNS-specific median progression-free survival; LM, leptomeningeal metastasis; HER2, human epidermal receptor 2; CSF, cerebrospinal fluid; MRI, magnetic resonance imaging; ECOG, Eastern Cooperative Oncology Group; ADC, antibody-drug conjugate T-DXd, trastuzumab-deruxtecan; T-DM1, trastuzumab-emtansine; IV, intravenous; IT, intrathecal.

**Quality (risk of bias) assessment:** To assess the methodological quality of individual studies included in the meta-analysis, we used a previously described tool that is adapted for evaluation of case reports and case series [14]. The tool includes five items that are derived from the Newcastle-Ottawa scale. These five items examine the selection and representativeness of cases and the ascertainment of outcomes and exposure, with each item scored one point if the information was specifically reported. The study was deemed to be of good quality (i.e. low risk of bias) when all five criteria were met (score of 5), of moderate quality when four criteria were met (score of 4), and of poor quality/high risk of bias when 3 or less criteria were met (score of 3 or less) [15].

**Statistical analyses:** We performed one-stage meta-analyses of pooled individual patient data from all included studies. Patient characteristics were compared between those who received intrathecal trastuzumab and those who did not, with Fisher's Exact test and Pearson's X^2^. The hazard ratio (HR) was used as the parameter of interest for OS and CNS-specific PFS. Cox proportional hazard models were used to determine the HR between groups of interest and its associated 95% confidence interval (CI). A multi-level mixed-effects Cox proportional hazards model, incorporating individual study as a random effect, was used to estimate the HR, its associated 95% CI, and P-value.

Multivariable Cox proportional hazards regression models were used to estimate adjusted OS and CNS-specific PFS (aOS and aPFS), also with a multi-level mixed-effects Cox proportional hazards regression model that incorporated individual study as a random effect. All variables with P < 0.05 in univariable analysis were incorporated into the initial multivariable model. We performed backward stepwise selection to remove insignificant variables. The final model included all variables with P < 0.05. For aOS, the initial multivariable model included geographical location (Europe), patient sample size in the study, age, hormone receptor status, lines of therapy in the metastatic setting, Eastern Cooperative Oncology Group (ECOG) status, treatment with neratinib and treatment with T-DXd versus T-DM1. For CNS-specific aPFS, the initial model included geographical location (Asia), patient sample size in the study, lines of therapy in the metastatic setting, status of prior HER2-targeted therapy, ECOG status, diagnosis with MRI, treatment with T-DXd, treatment with lapatinib, and treatment with T-DXd versus T-DM1. For both CNS-specific aPFS and aOS, this left only ECOG status as the only statistically significant variable in the multivariable model. We subsequently performed a sensitivity analysis by adding our variable of interest (IT versus IV/oral administration of HER2-targeted therapies) to the model, to obtain our final multivariable model. We tested the proportional hazards assumption by plotting the Schoenfeld residuals for each univariable and multivariable analysis, and they appeared random. Survival curves were visualized and evaluated with the Kaplan-Meier method and the log-rank test. Statistical analyses were performed with STATA v17 (StataCorp LLC, College Station, Texas, USA).

Correlation analyses between CNS-specific PFS and OS were performed with linear regression and Pearson's X^2^. When performance status was presented as Karnofsky Performance Status score, it was converted to ECOG using the previously described conversion scale [16].

**Patient data:** For the patients included in this study that were not previously included in other published reports, patients provided written consent for their medical records to be searched and included in this study in an anonymized fashion as case reports, in concordance with the Declaration of Helsinki.

## 3 Results

### 3.1 Characteristics of included studies and patients

**Fig. 1:**
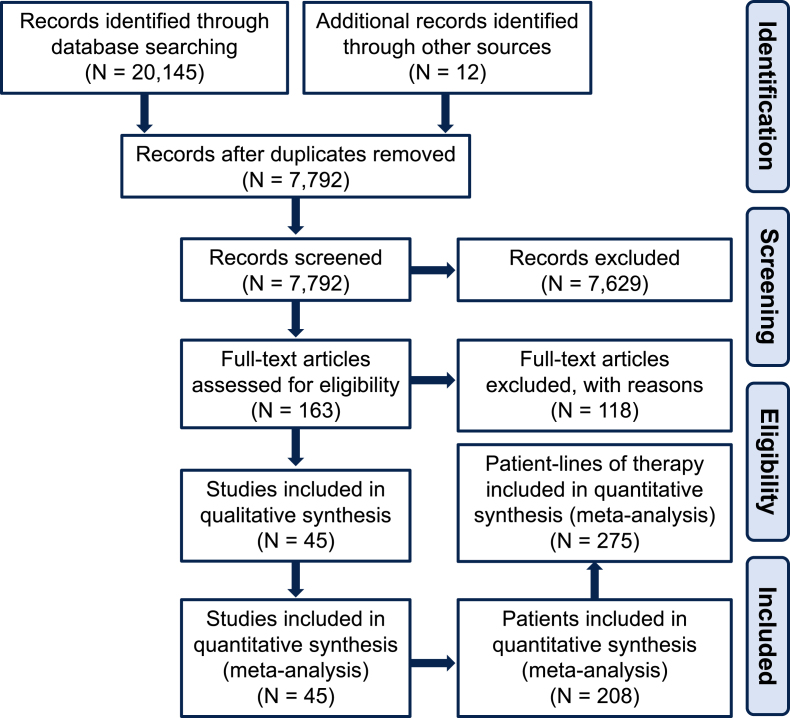
Preferred Reporting Items for Systematic Reviews and Meta-Analyses diagram demonstrating search and inclusion of studies for meta-analysis.

We identified 7780 potentially eligible articles in our search. After screening these articles, removal of ineligible articles and addition of studies from authors’ files, a total of 45 publications were included in our review (Appendix 2). This consisted of a total of 208 patients with HER2+ BC LM (Table 1) who received a total of 275 patient-lines of therapy for the treatment of LM (Fig. 1). A risk of bias assessment was also performed for all studies included in the meta-analysis on a 5-point scale (Supplemental Fig. S1).

Of the 275 patient-lines of therapy in our cohort, 92 received IT trastuzumab and 183 received regimens that included oral or IV HER2-targeted therapy (Table 1). When comparing clinical characteristics of the IT and no-IT cohorts, we observe that the no-IT cohort is enriched in patients of older age (P < 0.05), patients who were hormone receptor positive (P < 0.05), patients who were more likely to have concurrent extracranial metastases (P < 0.05), patients who were less likely to receive concurrent chemotherapy alongside HER2-targeted therapy (P < 0.001), and patients who were more likely to receive RT (P < 0.05). Patients in the IT cohort were more likely to have been reported in prospective studies (P < 0.001) and to be patients who had received fewer lines of therapy in the metastatic setting (P < 0.05).

### 3.2 Characteristics associated with OS and CNS-specific PFS

In the entire population, mOS and median CNS-specific PFS (mPFS) in the cohort was 14.3 and 6.0 months, respectively (Table 2, Table 3). In the patients where CNS-specific PFS was available from the source publication, we observe significant correlation between CNS-specific PFS and OS (Pearson's R^2^ = 0.63, P < 0.0001; Supplemental Fig. S2A). This significant correlation remains when analyses are restricted to patients who had documented death, or whose progression was not caused by death (Supplemental Fig. S2 B-D, Supplemental Table S2).

The following variables were associated with both shortened OS and CNS-specific PFS in univariable analysis: having received 2 or more lines of systemic therapy for metastatic disease (HR = 1.8, 95% CI: 1.2–2.8, P < 0.01 and HR = 1.6, 95% CI: 1.1–2.3, P < 0.05, respectively), ECOG performance status of 2 or greater (HR = 2.2, 95% CI: 1.5–3.2, P < 0.001, and HR = 1.9, 95% CI: 1.3–2.7, P = 0.001) and deriving from a study that included more than or equal to 5 patients (HR = 1.8, 95% CI: 1.2–2.6, P < 0.01 and HR = 1.7, 95% CI: 1.2–2.3 P < 0.01) (Table 2, Table 3). Age greater than or equal to 60 (HR = 1.7, 95% CI: 1.2–2.3, P < 0.001), hormone receptor positive status (HR = 3.3, 95% CI: 1.4–8.0, P < 0.05) and having received neratinib (HR = 2.1, 95% CI: 1.0–4.4, P < 0.05) were associated with shortened OS. Having received prior HER2-targeted therapy (HR = 3.6, 95% CI: 1.3–9.9, P < 0.05) was associated with shortened CNS-specific PFS, while diagnosis by MRI (HR = 0.68, 95% CI: 0.47–0.98, P < 0.05), receiving T-DXd (HR = 0.21, 95% CI: 0.07–0.58, P < 0.01) and originating from Asia (HR = 0.20, 95% CI: 0.06–0.97, P < 0.05) were associated with prolonged CNS-specific PFS.

**Fig. 2:**
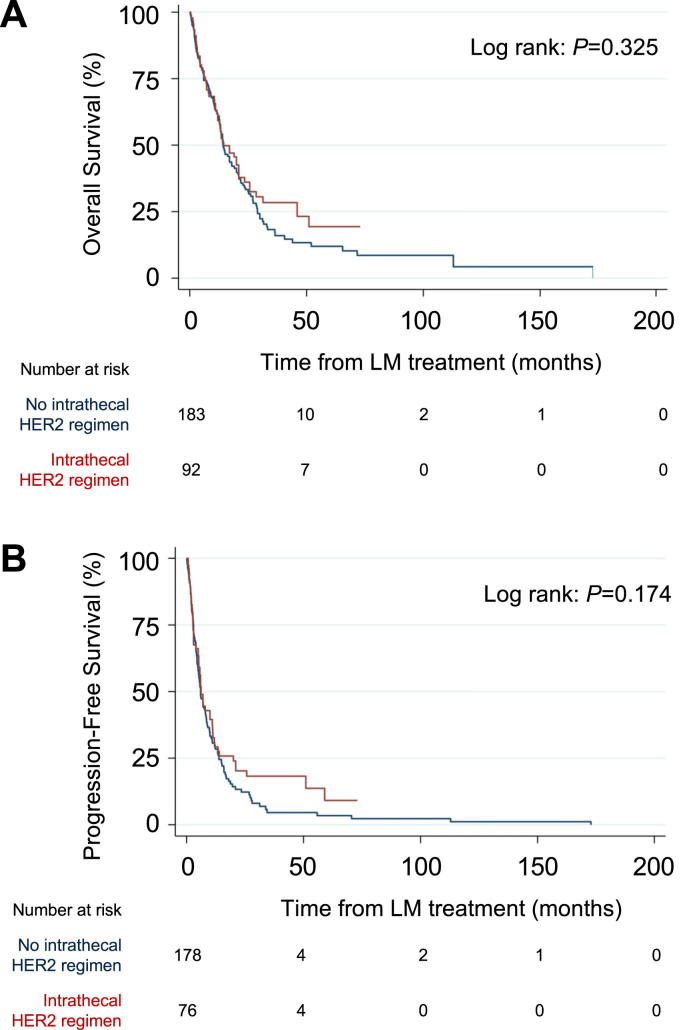
**Comparison of routes of administration of anti-HER2 therapy.** (A) OS and (B) CNS-specific PFS of patients who received intrathecal versus no intrathecal administration of a HER2-targeted regimen. P-values calculated with Log-Rank test.

In univariable analyses, IT trastuzumab was not associated with prolonged or shortened OS (HR = 0.92, 95% CI: 0.63–1.30, P = 0.66) or CNS-specific PFS (HR = 0.81, 95% CI: 0.60–1.1, P = 0.18) (Table 2, Table 3, Fig. 2A–B).

In multivariable analyses, IT trastuzumab was not independently associated with differential OS (HR = 1.5, 95% CI: 0.69–3.1, P = 0.33) or CNS-specific PFS (HR = 1.3, 95% CI: 0.61–2.6, P = 0.54). Meanwhile, ECOG performance status remained independently associated with differential OS and CNS-specific PFS in the final multivariable model (HR = 2.2, 95% CI: 1.5–3.1, P < 0.001 and HR = 1.9, 95% CI: 1.3–2.8, P = 0.001, respectively). ECOG status was not associated with route of trastuzumab delivery (P > 0.40) (Supplemental Table S3).

### 3.3 Comparing anti-HER2-targeted therapies for BC LM

We explored whether different categories of anti-HER2 therapeutics are associated with differential outcomes. We observe no significant difference in OS and CNS-specific PFS between regimens that included monoclonal antibody-based agents (trastuzumab, trastuzumab + pertuzumab, T-DM1, T-DXd) versus those that exclusively employed HER2 tyrosine kinase inhibitors (TKI; lapatinib, tucatinib, neratinib; Supplemental Fig. S3 A-B) or whether chemotherapy was added to anti-HER2-targeted therapies (Supplemental Fig S3 C-D). Moreover, the route of chemotherapy administration (IT, IV/oral or IT and IV/oral) was not associated with significant differences in OS or CNS-specific PFS (Supplemental Fig. S3 E-F).

Next, we examined whether individual agents are associated with prolonged OS and CNS-specific PFS. Trastuzumab, pertuzumab, and T-DM1 were not associated with differential outcomes (Supplemental Fig. S4 A-F), and lapatinib was associated with prolonged CNS-specific PFS (P = 0.024) but not OS (P = 0.094) compared to other HER2-targeted therapies (Supplemental Fig. S4 G-H).

**Fig. 3:**
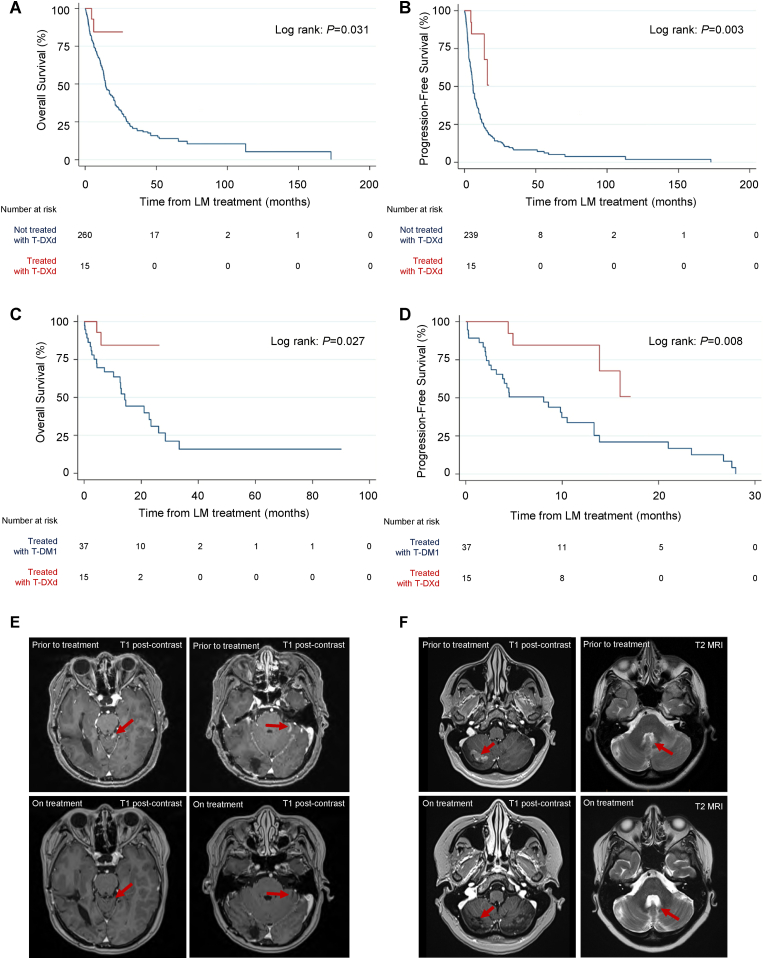
**Comparison of trastuzumab deruxtecan (TDXd) to other HER2-targeted therapies for breast cancer leptomeningeal metastases.** (A) OS and (B) CNS-specific PFS of patients who received treatment with T-DXd compared to those who did not. (C) OS and (D) CNS-specific PFS of patients who received treatment with T-DXd versus T-DM1. (E) T1 post-contrast MRIs obtained from a patient before and while on treatment with T-DXd. Left and right images represent unique views that demonstrate reduction in size of leptomeningeal lesions while on treatment. (F) T1 post-contrast (left) and T2 (right) MRIs obtained from a second patient before and while on treatment with T-DXd. Left (T1 post-contrast) images demonstrate reduction in size of leptomeningeal lesions while on treatment. Right (T2 MRI) images demonstrate improvement in mass effect on the fourth ventricle. The patients presented in (E) and (F) both demonstrated profound clinical improvements, one of which remains on treatment, with response ongoing. Red arrows point to areas of interest to compare in pre- and on-treatment MRIs. P-values calculated with Log-Rank test. (For interpretation of the references to colour in this figure legend, the reader is referred to the Web version of this article.)

Treatment with T-DXd was associated with prolonged OS (P < 0.05) and prolonged CNS-specific PFS (P < 0.01) (Fig. 3A–B). Furthermore, T-DXd demonstrated superior OS (P < 0.05) and CNS-specific PFS (P < 0.01) compared to T-DM1, another antibody drug conjugate (Fig. 3 C-D). Of the 15 patients treated with T-DXd who were included in the cohort, two are previously unpublished patients from our center. Both of these patients were treated with single agent T-DXd in the absence of surgery or RT for HER2+ BC LM and experienced profound clinical and image-based responses to treatment in their leptomeningeal lesions (Fig. 3 E-F). Both patients exhibited impressive responses lasting 16 months, one of which remains on treatment with ongoing treatment response.

### 3.4 Quality assessment

The majority of the patients included in this analysis were reported in retrospective studies. These patients may be subject to greater bias than patients identified from prospective studies. However, in our cohort, we observe no difference in OS or CNS-specific PFS between patients identified from retrospective versus prospective studies (HR = 1.5, 95% CI: 0.77–2.7, P = 0.25 and HR = 0.60, 95% CI: 0.19–1.9, P = 0.40, respectively) (Table 2, Table 3, Supplemental Fig. S5 A-B).

We next classified studies according to their risk of bias using a 5-point score that was adapted from the Newcastle-Ottawa scale [14]. Studies with a risk of bias (ROB) of 3 or less were classified as high risk of bias, while studies with an ROB of 4 or 5 were considered to have a moderate to low risk of bias. When comparing patients extracted from studies of moderate to low versus high risk of bias, we observe no significant differences in OS and CNS-specific PFS (HR = 1.1, 95% CI: 0.66–1.8, P = 0.70 and HR = 0.95, 95% CI: 0.60–1.5, P = 0.82, respectively) (Table 2, Table 3, Supplemental Fig. S5 C-D). We also observe no difference in outcomes of IT versus non-IT treated patients when only including those from studies with moderate to low risk of bias in the analysis (Supplemental Fig S5 E-F).

Furthermore, it has been previously shown that BC LM patients with spinal cord involvement experience worse prognosis compared to those who have brain-only disease [17]. For this reason, we explored whether patients with spinal cord involvement of their LM were more likely to derive benefit from IT therapy. No significant difference in OS was observed between patients with spinal versus brain-only LM (P = 0.8), while there was a non-significant trend towards prolonged CNS-specific PFS among patients with spinal cord involvement treated with IT HER2-targeted therapy (P = 0.060) (Supplemental Fig. S5 G-H).

## 4 Discussion

We initiated this study because HER2-targeted therapy is routinely used in patients with BC LM despite these patients not being included in any of the randomized controlled trials studying these agents. Therefore, high quality data on the efficacy of these HER2-directed therapies for patients with LM is lacking. By extracting data from 45 publications, corresponding to 208 patients and 275 lines of HER2-targeted therapy, we developed the largest cohort of treated HER2+ BC LM patients that has been compiled to date.

Our dataset demonstrates that HER2-targeted therapies have clinical activity in the setting of BC LM, with several patients experiencing durable and prolonged treatment responses. We identified no statistically significant difference in OS or CNS-specific PFS when HER2-targeted therapy is introduced intrathecally or intravenously. Several biological reasons can explain this finding. It is possible that IV trastuzumab reaches the subarachnoid space in sufficient concentrations to effectively treat LM in patients with trastuzumab-sensitive disease [18], and that the apparent resistance of BC LM to trastuzumab is largely mediated by the fact that the overwhelming majority of these patients have already received IV trastuzumab in prior lines of therapy (Table 1). It has been previously described that IV trastuzumab reaches significantly higher concentrations in the CSF of patients with LM and/or those who received WBRT compared to non-LM/non-WBRT patients [18]. However, these studies have also demonstrated that the trastuzumab concentrations in CSF are still an order of magnitude lower than serum concentrations even for LM patients receiving WBRT. Despite this, it is plausible that the microenvironmental concentrations at the site of LM lesions harboring local blood–CSF–barrier disruption approach serum levels that are sufficient to exert activity [18,19]. Furthermore, it has been demonstrated that IT trastuzumab rapidly distributes out of the CSF and into the serum, quickly negating any LM-specific efficacy that may exist with IT administration [20].

An additional benefit of employing IV over IT trastuzumab is that it would be expected to elicit greater activity for LM patients who have concurrent systemic disease. Indeed, 81% and 60% of patients who received IV/oral only and IT regimens in our dataset had extracranial metastases at the time of treatment, respectively (Table 1).

While no prospective trials of IV trastuzumab for BC LM have been published, two single-armed trials assessing the efficacy of IT trastuzumab in patients with HER2+ BC LM have been reported in the past year [6,7]. The phase II trial of IT trastuzumab (150 mg once weekly) in 19 patients with HER2+ BC with LM demonstrated a CNS-specific mPFS of 5.9 months and a mOS of 7.9 months [6]. Another phase I/II study of IT trastuzumab (80 mg twice weekly) in 26 HER2+ BC with LM demonstrated a mOS of 10.5 months [7]. Both studies did not describe the extracranial disease burden experienced by patients in their cohorts. However, we observe in our cohort that patients with extracranial metastases trended towards experiencing shorter OS but not CNS-specific PFS (Table 2, Table 3), suggesting that at least a subset of LM patients succumb to extracranial disease rather than their LM. We do, however, observe a significant correlation between CNS-specific PFS and OS in our cohort (Supplemental Fig. S2). This suggests that CNS-specific PFS has the potential to serve as a useful surrogate endpoint for this patient population with further refinement of standardized criteria to define LM response and progression [10,21,22].

Although we were not able to obtain individual patient data from these two recent trials and were thus unable to include them in this meta-analysis, the inclusion of these data would not alter our observation that IV trastuzumab is non-inferior to IT trastuzumab. This is because the mOS of 7.9 and 10.5 months in these two studies is shorter than the mOS of 14.5 months in the group of patients who received IT trastuzumab described herein [6,7].

The mOS in our cohort of patients with LM is longer than that generally cited in the literature [1]. Moreover, in our cohort, mOS and CNS-specific mPFS are shorter for patients included in larger studies (Table 2, Table 3). These observations can likely be attributed to publication bias, in that patients selected for publication in case reports and series experienced exceptional responses to treatment. Since this bias applies both to patients who received IT or IV treatment, it is unlikely that it would impact the key results described herein.

Beyond the lack of evidence demonstrating efficacy of IT over IV trastuzumab for HER2+ BC LM, a number of complications are associated with IT administration, such as drug-induced aseptic meningitis (DIAM) and infection of the IT reservoir through which the agents are administered. DIAM is a relatively uncommon complication of IT administration of trastuzumab, having been reported in two case reports across the literature [23,24]. However, 5%–8% of patients with an Ommaya reservoir experience Ommaya reservoir-related infections [25,26], a complication which is associated with prolonged hospital admissions and a mortality rate of approximately 10%. While the two aforementioned prospective studies demonstrate encouraging safety data for IT trastuzumab in their limited cohorts, it is clear that this approach encompasses additional risks of adverse events that are not present with IV therapy. For this reason, IT trastuzumab for HER2+ BC LM should require an additional burden of proof-of-efficacy before it is implemented outside of a clinical trial setting. While the window of opportunity for such a randomized-controlled trial has existed for several years, it is rapidly closing with novel agents such as T-DXd and tucatinib poised to be used in a growing number of HER2+ BC LM patients.

Despite the fact that we include only a small subgroup of 15 patients treated with T-DXd in this study, we were nonetheless able to observe a significant survival advantage with this agent over the rest of the population, and specifically against T-DM1 (Fig. 3). These results are in line with the recent TUXEDO-1 study demonstrating impressive efficacy of T-DXd for HER2+ BC patients with parenchymal brain metastases, and a recent publication by Alder et al. describing a case series of BC LM patients treated with T-DXd [27,28]. This sets the stage for future studies assessing the efficacy of T-DXd specifically for LM. Indeed, the ongoing DEBBRAH trial includes a cohort specifically for patients with HER2+ BC LM who will be treated with T-DXd [29]. T-DXd may have additional utility as a treatment for BC patients with HER2-low LM and HER2-mutant non-small cell lung cancer LM patients, given the positive results of the DESTINY-04 [30] and DESTINY-Lung01 trials [31].

Tucatinib is also a promising molecule under investigation for the treatment of HER2+ BC LM. We were limited in this study in that we were only able to acquire data that met inclusion criteria from 6 patients treated with tucatinib. However, preliminary data assessing the safety and efficacy of tucatinib, trastuzumab and capecitabine for HER2+ BC LM, following the positive results of this same regimen for the treatment of parenchymal brain metastases in the HER2CLIMB study, are encouraging [10,32,33].

Beyond HER2-targeted therapies, immunotherapy represents another promising treatment modality for this patient population [34,35], with IT administration of nivolumab being actively studied for LM [36]. A novel approach making use of bi-specific antibodies (HER2Bi) armed activated T-cells (HER2 BATs) was investigated in a recent phase I trial (NCT03661424). While this trial was terminated due to slow study accrual, further studies are required to assess the efficacy of HER2 BATs for the treatment of LM. Moreover, while none of the patients from our dataset received proton craniospinal irradiation, this novel RT approach has demonstrated efficacy for patients with solid tumor LM, representing another encouraging component of the treatment armamentarium for HER2+ BC LM in development [37].

There are several limitations associated with our study. Many of the patients included in this meta-analysis are derived from case reports and retrospective case series, resulting in imbalances in some of the patient characteristics between those who received IT versus non-IT therapy. While we have taken measures to control for this bias, such as performing quality assessment and performing extensive subset analyses, there is no alternative for a well-designed randomized controlled trial to directly compare HER2-targeted agents and their route of administration. In addition, we are limited by publication bias, whereby patients who experienced better than expected responses to therapy were more likely to be published in the literature. For this reason, the CNS-specific mPFS and mOS we observe herein of 6 and 14.3 months, respectively, are overestimations of the outcomes seen in real-world studies of patients with HER2+ BC LM [38]. Furthermore, data regarding CNS-specific PFS must be considered with caution as the evaluation of LM response and LM progression is highly challenging and could vary across studies [21].

Together, the results of this study demonstrate that HER2-targeted therapy is similarly active in patients with HER2+ BC LM regardless of the route of administration. T-DXd demonstrates an encouraging signal of efficacy in a small subgroup of patients. Prospective and randomized studies are warranted to define its role in the management of HER2+ BC LM.

## Author contributions

**Conception & Design of the study:** AML, AQ, AANR, NB, MD. **Acquisition of Data:** AML, SMM, AD, IT, EF, GG, VG, AP, SH, WJ, JS, MPHVDB, ND, FP, ZL, AM, AS, RS, LP, AANR, NB, MD. **Analysis and interpretation of data:** AML, NB, MD. **Drafting the article:** AML, MD. **Revising the article:** AML, SMM, AQ, AD, IR, EF, GG, AP, SH, WJ, JS, MPHVDB, ND, FP, ZL, AM, AS, RS, LP, AANR, NB, MD.

## Funding

MD is an awardee of a Vanier Canada Graduate Scholarship.

## Declaration of competing interest

The authors declare that they have no known competing financial interests or personal relationships that could have appeared to influence the work reported in this paper.

## Figures and Tables

**Table:** 

Total with duplicates	
Duplicates removed	
Total without duplicates	
Database	Ovid MEDLINE(R) ALL
Database time coverage	1946-present
Date searched	21 December 2021
Total	3646
Duplicates	
Database	Ovid Embase Classic + Embase
Database time coverage	1947-present
Date searched	21 December 2021
Total	6929
Duplicates	
Database	Cochrane Central Register of Controlled Trials (Wiley)
Database time coverage	n/a
Date searched	22 December 2021
Total	156
Duplicates	
Database	Scopus
Database time coverage	1970-present
Date searched	22 December 2021
Total	5560
Duplicates	
Database	Web of Science Core Collection (SCI-EXPANDED – 1900-present; SSCI – 1900-present; AHCI – 1975-present; CPCI–S – 1990-present; CPCI-SSH – 1990-present; BKCI–S – 2005-present; BKCI-SSH – 2005-present; ESCI – 2005-present; CCR-EXPANDED – 1985-present; IC – 1993-present)
Database time coverage	1900-present
Date searched	22 December 2021
Total	3854
Duplicates	

**Table:** 

**Author last name**	**Year**	**Reference**	**Number of patient lines of therapy**	**Number of patients**	**Data**
Baculi	2001	Baculi RH, Suki S, Nisbett J, Leeds N, Groves M. Meningeal carcinomatosis from breast carcinoma responsive to trastuzumab. J Clin Oncol. 2001 Jul 1; 19 (13):3297–8. https://doi.org/10.1200/JCO.2001.19.13.3297. PMID: Baculi RH, Suki S, Nisbett J, Leeds N, Groves M. Meningeal carcinomatosis from breast carcinoma responsive to trastuzumab. J Clin Oncol. 2001 Jul 1; 19 (13):3297–8. https://doi.org/10.1200/JCO.2001.19.13.3297. PMID: 11432901.	1	1	Data extracted by authors of this manuscript
Platini	2006	Platini C, Long J, Walter S. Meningeal carcinomatosis from breast cancer treated with intrathecal trastuzumab. Lancet Oncol. 2006 Sep; 7 (9):778–80. https://doi.org/10.1016/S1470-2045(0670864-6). PMID: 16945774.	3	1	Data extracted by authors of this manuscript
Stemmler	2006	Stemmler HJ, Schmitt M, Harbeck N, Willems A, Bernhard H, Lässig D, Schoenberg S, Heinemann V. Application of intrathecal trastuzumab (Herceptintrade mark) for treatment of meningeal carcinomatosis in HER2-overexpressing metastatic breast cancer. Oncol Rep. 2006 May; 15 (5):1373–7. https://doi.org/10.3892/or.15.5.1373. PMID: 16596213.	2	1	Data obtained by contacting authors
Stemmler	2007	Stemmler HJ, Schmitt M, Willems A, Bernhard H, Harbeck N, Heinemann V. Ratio of trastuzumab levels in serum and cerebrospinal fluid is altered in HER2-positive breast cancer patients with brain metastases and impairment of blood–brain barrier. Anti-Cancer Drugs: January 2007-vol 18 - Issue 1 - p 23–28 https://doi.org/10.1097/01.cad.0000236313.50833.ee	2	1	Data obtained by contacting authors
Mir	2008	Mir O, Ropert S, Alexandre J, Lemare F, Goldwasser F. High-dose intrathecal trastuzumab for leptomeningeal metastases secondary to HER-2 overexpressing breast cancer. Ann Oncol. 2008 Nov; 19 (11):1978–80. https://doi.org/10.1093/annonc/mdn654. Epub 2008 Oct 9. PMID: 18845838.	1	1	Data extracted by authors of this manuscript
Shojima	2008	Shojima K, Suzuki E, Saito K, Sekine S, Kitagawa D, Aruga T, Saji S, Kuroi K. Application of intrathecal trastuzumab for treatment of meningeal carcinomatosis in HER2-overexpressing metastatic breast cancer. Journal of Clinical Oncology 2008 26:15_suppl, 1138-1138.	1	1	Data extracted by authors of this manuscript
Stemmler	2008	Stemmler HJ, Mengele K, Schmitt M, Harbeck N, Laessig D, Herrmann KA, Schaffer P, Heinemann V. Intrathecal trastuzumab (Herceptin) and methotrexate for meningeal carcinomatosis in HER2-overexpressing metastatic breast cancer: a case report. Anticancer Drugs. 2008 Sep; 19 (8):832–6. https://doi.org/10.1097/CAD.0b013e32830b58b0. PMID: 18690096.	1	1	Data obtained by contacting authors
Bidard	2009	Bidard FC, Guilhaume MN, Gauthier H, Cottu PH, Diéras V, Pierga JY. Meningeal carcinomatosis in HER2-overexpressing breast cancers. J Neurooncol. 2009 Jun; 93 (2):287–8. https://doi.org/10.1007/s11060-008-9768-1. Epub 2009 Jan 13. PMID: 19139826.	5	5	Data extracted by authors of this manuscript
Ferrario	2009	Ferrario C, Davidson A, Bouganim N, Aloyz R, Panasci LC. Intrathecal trastuzumab and thiotepa for leptomeningeal spread of breast cancer. Ann Oncol. 2009 Apr; 20 (4):792–5. https://doi.org/10.1093/annonc/mdp019. Epub 2009 Feb 17. PMID: 19223574.	1	1	Data extracted by authors of this manuscript
Mego	2011	Mego M, Sycova-Mila Z, Obertova J, Rajec J, Liskova S, Palacka P, Porsok S, Mardiak J. Intrathecal administration of trastuzumab with cytarabine and methotrexate in breast cancer patients with leptomeningeal carcinomatosis. Breast. 2011 Oct; 20 (5):478–80. https://doi.org/10.1016/j.breast.2011.05.007. Epub 2011 Jun 23. PMID: 21700455.	2	2	Data extracted by authors of this manuscript
Oliveira	2011	Oliveira M, Braga S, Passos-Coelho JL, Fonseca R, Oliveira J. Complete response in HER2+ leptomeningeal carcinomatosis from breast cancer with intrathecal trastuzumab. Breast Cancer Res Treat. 2011 Jun; 127 (3):841–4. https://doi.org/10.1007/s10549-011-1417-2. Epub 2011 Mar 3. PMID: 21369716.	5	1	Data extracted by authors of this manuscript
Schwab Brandt	2012	Brandt D (2012). Intrathecal trastuzumab: 46 months and no progression. Community Oncology. 9.232–234.10.1016/j.cmonc.2012.01.005.	1	1	Data extracted by authors of this manuscript
Martens	2012	Martens J, Venuturumilli P, Corbets L, Bestul D. Rapid clinical and radiographic improvement after intrathecal trastuzumab and methotrexate in a patient with HER-2 positive leptomeningeal metastases. Acta Oncol. 2013 Jan; 52 (1):175–8. https://doi.org/10.3109/0284186X.2012.689857. Epub 2012 Jun 4. PMID: 22655969.	1	1	Data extracted by authors of this manuscript
Pardo	2012	Pardo J (2012). Intratechal trastuzumab in the treatment of neoplastic meningitis: three new cases.	3	3	Data extracted by authors of this manuscript
Hofer	2012	Hofer S, Mengele K, Stemmler HJ, Schmitt M, Pestalozzi B. Intrathecal trastuzumab: dose matters. Acta Oncol. 2012 Sep; 51 (7):955–6. https://doi.org/10.3109/0284186X.2012.673736. Epub 2012 Apr 23. PMID: 22524214.	1	1	Data obtained by contacting authors
Preusser	2013	Preusser ML, Berghoff AS, Furtner J, Dieckmann D, Bartsch R. (2013). Meningeosis carcinomatosa eines HER2-positiven Mammakarzinoms.	1	1	Data extracted by authors of this manuscript
Torres	2014	Torres S, Maralani P, Verma S. Activity of T-DM1 in HER-2 positive central nervous system breast cancer metastases. BMJ Case Rep. 2014 Aug 14; 2014:bcr2014205680. https://doi.org/10.1136/bcr-2014-205680. PMID: 25123575; PMCID: PMC4139549.	2	2	Data extracted by authors of this manuscript
Hofer	2015	Hofer S, Mengele K, Schmitt M, Pestalozzi B, Aebi S. Complement Activation and Rituximab Distribution in CNS NHL—Letter. Clin Cancer Res 15 January 2015; 21 (2): 490. https://doi.org/10.1158/1078-0432.CCR-14-0939	3	3	Data obtained by contacting authors
Le Rhun	2015	Le Rhun E, Taillibert S, Boulanger T, Zairi F, Bonneterre J, Chamberlain MC. Prolonged Response and Restoration of Functional Independence with Bevacizumab plus Vinorelbine as Third-Line Treatment for Breast Cancer-Related Leptomeningeal Metastases. Case Rep Oncol. 2015 Feb 12; 8 (1):72–7. https://doi.org/10.1159/000375293. PMID: 25848355; PMCID: PMC4361905.	2	1	Data extracted by authors of this manuscript
Lu	2015	Lu NT, Raizer J, Gabor EP, Liu NM, Vu JQ, Slamon DJ, Barstis JL. Intrathecal trastuzumab: immunotherapy improves the prognosis of leptomeningeal metastases in HER-2+ breast cancer patient. J Immunother Cancer. 2015 Sep 15; 3:41. https://doi.org/10.1186/s40425-015-0084-y. PMID: 26380087; PMCID: PMC4570757.	4	1	Data extracted by authors of this manuscript
Gulia	2016	Gulia S, Gupta S, Singh A. Intrathecal trastuzumab for leptomeningeal carcinomatosis in patients with human epidermal growth factor receptor 2 positive breast cancer. Indian J Med Paediatr Oncol. 2016 Jul–Sep; 37 (3):196–8. https://doi.org/10.4103/0971-5851.190354. PMID: 27688614; PMCID: PMC5027793.	2	2	Data extracted by authors of this manuscript
Jacot	2016	Jacot W, Pons E, Frenel JS, Guiu S, Levy C, Heudel PE, Bachelot T, D'Hondt V, Darlix A, Firmin N, Romieu G, Thezenas S, Dalenc F. Efficacy and safety of trastuzumab emtansine (T-DM1) in patients with HER2-positive breast cancer with brain metastases. Breast Cancer Res Treat. 2016 Jun; 157 (2):307–318. https://doi.org/10.1007/s10549-016-3828-6. Epub 2016 May 11. PMID: 27167986.	3	3	Data obtained by contacting authors
Koumarianou	2016	Koumarianou A, Kontopoulou C, Kouloulias V, Tsionou C. Durable Clinical Benefit of Pertuzumab in a Young Patient with BRCA2 Mutation and HER2-Overexpressing Breast Cancer Involving the Brain. Case Rep Oncol Med. 2016; 2016:5,718,104. https://doi.org/10.1155/2016/5718104. Epub 2016 Apr 18. PMID: 27195161; PMCID: PMC4852335.	5	1	Data extracted by authors of this manuscript
Lavaud	2016	Lavaud P, Rousseau B, Ajgal Z, Arrondeau J, Huillard O, Alexandre J, Hulin A, Goldwasser F. Bi-weekly very-high-dose lapatinib: an easy-to-use active option in HER-2-positive breast cancer patients with meningeal carcinomatosis. Breast Cancer Res Treat. 2016 May; 157 (1):191–2. https://doi.org/10.1007/s10549-016-3798-8. Epub 2016 Apr 22. PMID: 27106482.	1	1	Data extracted by authors of this manuscript
Lekovic	2016	Lekovic G, Drazin D, Mak AC, Schwartz MS. Cyberknife Radiosurgery and Concurrent Intrathecal Chemotherapy for Leptomeningeal Metastases: Case Report of Prolonged Survival of a HER-2+ Breast Cancer Patient Status-Post Craniospinal Irradiation. Cureus. 2016 Jan 7; 8 (1):e453. https://doi.org/10.7759/cureus.453. PMID: 26918221; PMCID: PMC4744073.	1	1	Data extracted by authors of this manuscript
Park	2016	Park WY, Kim HJ, Kim K, Bae SB, Lee N, Lee KT, Won JH, Park HS, Lee SC. Intrathecal Trastuzumab Treatment in Patients with Breast Cancer and Leptomeningeal Carcinomatosis. Cancer Res Treat. 2016 Apr; 48 (2):843–7. https://doi.org/10.4143/crt.2014.234. Epub 2015 Mar 2. PMID: 25761487; PMCID: PMC4843730.	2	2	Data extracted by authors of this manuscript
Pluchart	2016	Pluchart H, Jacquet E, Charlety D, Allenet B, Bedouch P, Mousseau M. Long-Term Survivor with Intrathecal and Intravenous Trastuzumab Treatment in Metastatic Breast Cancer. Target Oncol. 2016 Oct; 11 (5):687–691. https://doi.org/10.1007/s11523-016-0429-6. PMID: 27041112.	1	1	Data extracted by authors of this manuscript
Morikawa	2017	Morikawa A, Jordan L, Rozner R, Patil S, Boire A, Pentsova E, Seidman AD. Characteristics and Outcomes of Patients With Breast Cancer With Leptomeningeal Metastasis. Clin Breast Cancer. 2017 Feb; 17 (1):23–28. https://doi.org/10.1016/j.clbc.2016.07.002. Epub 2016 Jul 25. PMID: 27569275; PMCID: PMC5266701.	16	16	Data obtained by contacting authors
Bonneau	2018	Bonneau C, Paintaud G, Trédan O, Dubot C, Desvignes C, Dieras V, Taillibert S, Tresca P, Turbiez I, Li J, Passot C, Mefti F, Mouret-Fourme E, Le Rhun E, Gutierrez M. Phase I feasibility study for intrathecal administration of trastuzumab in patients with HER2 positive breast carcinomatous meningitis. Eur J Cancer. 2018 May; 95:75–84. https://doi.org/10.1016/j.ejca.2018.02.032. Epub 2018 Apr 7. PMID: 29635147.	16	16	Data extracted by authors of this manuscript
Figura	2018	Figura NB, Long W, Yu M, Robinson TJ, Mokhtari S, Etame AB, Tran ND, Diaz R, Soliman H, Han HS, Sahebjam S, Forsyth PA, Ahmed KA. Intrathecal trastuzumab in the management of HER2+ breast leptomeningeal disease: a single institution experience. Breast Cancer Res Treat. 2018 Jun; 169 (2):391–396. https://doi.org/10.1007/s10549-018-4684-3. Epub 2018 Feb 1. PMID: 29392582.	13	13	Data extracted by authors of this manuscript
Hofer	2018	Hofer S, Aebi S. Letter comments on EJC published article: Phase I feasibility study for intrathecal administration of trastuzumab in patients with HER2-positive breast carcinomatous meningitis. Eur J Cancer. 2018 Nov; 103:279–280. https://doi.org/10.1016/j.ejca.2018.07.317. Epub 2018 Sep 27. PMID: 30270111.	4	4	Data obtained by contacting authors
Ricciardi	2018	Ricciardi GRR, Russo A, Franchina T, Schifano S, Mastroeni G, Santacaterina A, Adamo V. Efficacy of T-DM1 for leptomeningeal and brain metastases in a HER2 positive metastatic breast cancer patient: new directions for systemic therapy - a case report and literature review. BMC Cancer. 2018 Jan 25; 18 (1):97. https://doi.org/10.1186/s12885-018-3994-5. PMID: 29370839; PMCID: PMC5784540.	1	1	Data extracted by authors of this manuscript
Figura	2019	Figura NB, Rizk VT, Mohammadi H, Evernden B, Mokhtari S, Yu HM, Robinson TJ, Etame AB, Tran ND, Liu J, Washington I, Diaz R, Czerniecki BJ, Soliman H, Han HS, Sahebjam S, Forsyth PA, Ahmed KA. Clinical outcomes of breast leptomeningeal disease treated with intrathecal trastuzumab, intrathecal chemotherapy, or whole brain radiation therapy. Breast Cancer Res Treat. 2019 Jun; 175 (3):781–788. https://doi.org/10.1007/s10549-019-05170-7. Epub 2019 Mar 11. PMID: 30859348.	18	18	Data extracted by authors of this manuscript
Matsuda	2019	Matsuda T, Iguchi E, Konishi E, Tokugawa T, Hamaoka A, Nakatsukasa K. [A Case of Breast Cancer with Parenchymal and Meningeal Central Nervous System Metastases Treated with Multimodality Therapy]. Gan To Kagaku Ryoho. 2019 Mar; 46 (3):463–465. Japanese. PMID: 30914585.	1	1	Data extracted by authors of this manuscript
Nakao	2019	Nakao T, Okuda T, Fujita M, Kato A. A case of leptomeningeal metastases of human epidermal growth factor receptor 2-positive breast cancer that responded well to lapatinib plus capecitabine. Surg Neurol Int. 2019 Jun 28; 10:131. https://doi.org/10.25259/SNI-106-2019. PMID: 31528467; PMCID: PMC6744731.	1	1	Data extracted by authors of this manuscript
Garcia	2020	García FJV, Carrión NP, de la Cruz-Merino L. Long-term complete response to intrathecal trastuzumab in a patient with leptomeningeal carcinomatosis due to her2- overexpressing breast cancer: Case report. Medicine (Baltimore). 2020 Jan; 99 (1):e18298. https://doi.org/10.1097/MD.0000000000018298. PMID: 31895768; PMCID: PMC6946348.	1	1	Data extracted by authors of this manuscript
Sallevelt	2020	Sallevelt BTGM, Teunis T, Agterof MJ, van den Broek MPH. Extravasation of an antibody-drug conjugate: A case report of epidermal necrosis after trastuzumab-emtansine extravasation. J Clin Pharm Ther. 2020 Aug; 45 (4):832–835. https://doi.org/10.1111/jcpt.13148. Epub 2020 May 15. PMID: 32412114; PMCID: PMC7383643.	2	2	Data obtained by contacting authors
Morikawa	2020	Morikawa A, de Stanchina E, Pentsova E, Kemeny MM, Li BT, Tang K, Patil S, Fleisher M, Van Poznak C, Norton L, Seidman AD. Phase I Study of Intermittent High-Dose Lapatinib Alternating with Capecitabine for HER2-Positive Breast Cancer Patients with Central Nervous System Metastases. Clin Cancer Res. 2019 Jul 1; 25 (13):3784–3792. https://doi.org/10.1158/1078-0432.CCR-18-3502. Epub 2019 Apr 15. PMID: 30988080; PMCID: PMC6773251.	3	3	Data obtained by contacting authors
Higashiyama	2021	Higashiyama N, Nangia J, Shafaee MN, Chen N, Michael BL, Rimawi M, Hoyos V. Dose-reduced trastuzumab deruxtecan can be safely used in liver failure and active leptomeningeal metastases. Curr Probl Cancer Case Rep. 2020 Dec 15; 2:100,034. https://doi.org/10.1016/j.cpccr.2020.100034. Epub 2020 Oct 16. PMID: 34505091; PMCID: PMC8425325.	1	1	Data extracted by authors of this manuscript
Pellerino	2021	Pellerino A, Soffietti R, Bruno F, Manna R, Muscolino E, Botta P, Palmiero R, Rudà R. Neratinib and Capecitabine for the Treatment of Leptomeningeal Metastases from HER2-Positive Breast Cancer: A Series in the Setting of a Compassionate Program. Cancers (Basel). 2022 Feb 25; 14 (5):1192. https://doi.org/10.3390/cancers14051192. PMID: 35267501; PMCID: PMC8909342.	10	10	Data obtained by contacting authors
Yan	2022	Yan F, Rinn KJ, Kullnat JA, Wu AY, Ennett MD, Scott EL, Kaplan HG. Response of Leptomeningeal Metastasis of Breast Cancer With a HER2/neu Activating Variant to Tucatinib: A Case Report. J Natl Compr Canc Netw. 2022 Apr 11; 20 (7):745–752. https://doi.org/10.6004/jnccn.2022.7006. PMID: 35405660.	1	1	Data extracted by authors of this manuscript
Smith	2022	Smith PD, Bhenderu LS, Kommuri S, Fleener EE, Hoover JM. Treatment of Leptomeningeal Carcinomatosis Following Treatment of Cerebellar Metastasis of HER2+ (Human Epidermal Growth Factor Receptor 2 Positive) Breast Cancer: Case Report and Review of Literature. Cureus. 2022 Apr 10; 14 (4):e24008. https://doi.org/10.7759/cureus.24008. PMID: 35547416; PMCID: PMC9090228.	1	1	Data extracted by authors of this manuscript
Ratosa	2022	Ratosa I, Dobnikar N, Bottosso M, Dieci MV, Jacot W, Pouderoux S, Ribnikar D, Sinoquet L, Guarneri V, Znidaric T, Darlix A, Griguolo G. Leptomeningeal metastases in patients with human epidermal growth factor receptor 2 positive breast cancer: Real-world data from a multicentric European cohort. Int J Cancer. 2022 Oct 15; 151 (8):1355–1366. https://doi.org/10.1002/ijc.34135. Epub 2022 Jun 25. PMID: 35666525; PMCID: PMC9540903.	61	45	Data obtained by contacting authors
Griguolo	2022	Griguolo G, Pouderoux S, Dieci MV, Jacot W, Bourgier C, Miglietta F, Firmin N, Conte P, Viala M, Guarneri V, Darlix A. Clinicopathological and Treatment-Associated Prognostic Factors in Patients with Breast Cancer Leptomeningeal Metastases in Relation to Tumor Biology. Oncologist. 2018 Nov; 23 (11):1289–1299. https://doi.org/10.1634/theoncologist.2018-0200. Epub 2018 Aug 17. PMID: 30120164; PMCID: PMC6291333.	47	23	Data obtained by contacting authors
	2022		3	2	
Alder	2023	Alder L, Trapani D, Bradbury C, Van Swearingen AED, Tolaney SM, Khasraw M, Anders CK, Lascola CD, Hsu L, Lin NU, Sammons S. Durable responses in patients with HER2+ breast cancer and leptomeningeal metastases treated with trastuzumab deruxtecan. NPJ Breast Cancer. 2023 Mar 30; 9 (1):19. https://doi.org/10.1038/s41523-023-00519-0. PMID: 36997605.	18	8	Data extracted by authors of this manuscript
		Total	275	208	
		Data obtained through data extraction by authors of this manuscript	117	93	
		Data obtained by contacting authors	158	115	
		Percent data obtained through data extraction by authors of this manuscript	42.5454545454546	44.7115384615385	
		Percent data obtained by contacting authors	57.4545454545455	55.2884615384615	
		Number of patients not obtained		343	
		Percent number of patients we obtained compared to total number of patients		37.7495462794918	

**Table:** 

**Title**	**Authors**	**First Author Last Name**	**Year of Study**	**Type of study**	**Patient sample size**
Leptomeningeal disease and breast cancer: the importance of tumor subtype	Abouharb, S. and Ensor, J. and Loghin, M. E. and Katz, R. and Moulder, S. L. and Esteva, F. J. and Smith, B. and Valero, V. and Hortobagyi, G. N. and Melhem-Bertr and t, A.	Abouharb	2014	Retrospective study	56
Intra-CSF trastuzumab in patients with neoplastic meningitis from breast cancer or primary brain tumors	Allison, D. L. and Glantz, M. and Werner, T. L. and Kirkegaard, S. L. and Murdock, K. and Jensen, R.	Allison	2009	Case series	4
Favourable outcome of patients with breast cancer brain metastases treated with dual HER2 blockade of trastuzumab and pertuzumab	Bergen, E. S. and Binter, A. and Starzer, A. M. and Heller, G. and Kiesel, B. and Tendl-Schulz, K. and Bago-Horvath, Z. and Furtner, J. and Leitner, J. and Exner, R. and Fitzal, F. and Dieckmann, K. and Widhalm, G. and Preusser, M. and Berghoff, A. S. and Bartsch, R.	Bergen	2021	Retrospective study	3
Safety and efficacy of intraventricular biologic agents as part of a multi-agent intraventricular treatment regimen for patients with neoplastic meningitis	Bernstein, A. and Mrowczynski, O. and Strowd, R. E. and Cream, L. and Ruda, R. and Jeyapalan, S. and Eby, R. and Black, D. and Patrikidou, A. and Hofer, S. and Ferreri, A. and Glantz, M.	Bernstein	2017	Case series	13
Multicentric, open-label, single-arm phase II study with oral lapatinib in combination with oral capecitabine plus intrathecally administered liposomal cytarabine for the treatment of meningeal metastases (NM) in HER2-positive breast cancer patients	Bischoff, J.	Bischoff	2010	Prospective trial	34
Treatment options of long term survivors with leptomeningeal metastases and breast cancer	Chaul-Barbosa, C. and Morikawa, A. and Patil, S. and Boire, A. and Jordan, L. and Rozner, R. and Seidman, A. and Pentsova, E.	Chaul-Barbosa	2016	Retrospective study	16
Final results of the phase I “HIT” study: A multicenter phase I-II study evaluating trastuzumab administered by intrathecal injection for leptomeningeal meningitis of HER2+ metastatic breast cancer (MBC)	Gutierrez, M. and Fourme, E. M. and Le Rhun, E. and Tredan, O. and Dieras, V. and Tresca, P. and Mefti, F. and Turbiez, I. and Taillibert, S. and Desvignes, C. and Paintaud, G.	Gutierrez	2015	Prospective trial	16
The therapeutic possibility of intrathecal administration of trastuzumab for the carcinomatous meningitis of HER2-positive metastatic breast cancer: The low penetration of trastuzumab into the cerebrospinal fluid via intravenous administration	Honda, Y. and Yamashita, T. and Iwamoto, N. and Goto, R. and Idera, N. and Horiguchi, K. and Miyamoto, H. and Aruga, T. and Yamada, R. and Kuroi, K.	Honda	2017	Case series	7
Treatment and prognosis of leptomeningeal disease secondary to metastatic breast cancer: A single-center experience	Kingston, B. and Kayhanian, H. and Brooks, C. and Cox, N. and Chaabouni, N. and Redana, S. and Kalaitzaki, E. and Smith, I. and O'Brien, M. and Johnston, S. and Parton, M. and Noble, J. and Stanway, S. and Ring, A. and Turner, N. and Okines, A.	Kingston	2017	Retrospective study	48
Intrathecal (IT) traztuzumab (T) for the treatment of leptomeningeal metastases (LM) in patients (PTS) with human epidermal growth factor receptor 2-positive (HER2+) cancer: A multicenter phase 1/2 study	Kumthekar, P. and Gradishar, W. and Lin, N. and Pentsova, E. and Groves, M. and Jeyapalan, S. and Melisko, M. and Grimm, S. and Lassman, A. B. and Raizer, J.	Kumthekar	2018	Prospective trial	34
Antigen Mass May Influence Trastuzumab Concentrations in Cerebrospinal Fluid After Intrathecal Administration	Le Tilly, O. and Azzopardi, N. and Bonneau, C. and Desvignes, C. and Oberkampf, F. and Ezzalfani, M. and Ternant, D. and Turbiez, I. and Gutierrez, M. and Paintaud, G.	Le Tilly	2021	Case series	21
Response to ado-trastuzumab emtansine according to RANO criteria in central nervous system metastases of HER2 positive breast cancers	Mailliez, A. and Girard, E. and Boulanger, T. and Giraud, C. and Bonneterre, J. and Le Rhun, E.	Mailliez	2016	Retrospetive study	1
TBCRC049: A phase II non-randomized study to assess the safety and efficacy of the combination of tucatinib and trastuzumab and capecitabine for treatment of leptomeningeal metastases in HER2 positive breast cancer TBCRC049: A phase II non-randomized study to assess the safety and efficacy of the combination of tucatinib and trastuzumab and capecitabine for treatment of leptomeningeal metastases in HER2 positive breast cancer	Murthy, R. K. and O'Brien, B. J. and Hess, K. R. and Navin, N. and Johnson, J. and Gule-Monroe, M. and Leone, J. P. and Specht, J. and Melisko, M. and Morikawa, A. and Storniolo, A. M. and Brufsky, A. and Pohlmann, P. R. and Park, D. M. and Park, B. H. and Krop, I. and Lin, N. U. and Wolff, A. and Forerro-Torres, A. and Stringer-Reasor, E.	Murthy	2020	Prospective trial	Trial ongoing: currently 15 patients, accrual of 30 patients
Determinants of prolonged survival for breast cancer patient groups with leptomeningeal metastasis (LM)	Niwinska, A. and Pogoda, K. and Michalski, W. and Kunkiel, M. and Jagiello-Gruszfeld, A.	Niwinska	2018	Retrospective study	33
Intrathecal (IT) trastuzumab in leptomeningeal and central nervous system (CNS) metastases from HER2+ breast cancer (BC): What if we could bypass the blood-brain barrier (BBB)	Oliveira, M. and Braga, S. and Passos-Coelho, J. L. and Oliveira, J.	Oliveira	2010	Case report	1
Prospective evaluation of cerebrospinal fluid circulating tumor cells (CSF CTC) in patients with HER2 positive cancers and leptomeningeal metastases receiving treatment with intrathecal trastuzumab	Pentsova, E. and Malani, R. and Fleisher, M. and Lin, X. and Omuro, A. and Groves, M. and Lin, N. and Melisko, M. and Lassman, A. and Jeyapalan, S. and Boire, A. and DeAngelis, L. and Raizer, J.	Pentsova	2018	Case series	14
Phase I trial of intrathecal trastuzumab in HER2 positive leptomeningeal metastases	Raizer, J. and Pentsova, E. and Omuro, A. and Lin, N. and Nayak, L. and Quant, E. and Kumthekar, P.	Raizer	2014	Prospective trial	13
[A case of advanced breast cancer with meningeal carcinomas and orbital metastasis successfully treated with multi-disciplinary therapy]	Sakurai, K. and Amano, S. and Enomoto, K. and Matsuo, S.	Sakurai	2006	Case report	1
Implication of breast cancer phenotype for patients with leptomeningeal carcinomatosis	Torrejon, D. and Oliveira, M. and Cortes, J. and Sanchez-Olle, G. and Gomez, P. and Bellet, M. and Saura, C. and Peg, V. and Rovira, A. and Di Cosimo, S.	Torrejon	2012	Retrospective study	10
Safety and activity of intra-CSF trastuzumab in patients with neoplastic meningitis from breast cancer or primary brain tumors	Zalatimo, O. and Weston, C. and Zoccoli, C. and Glantz, M.	Zalatimo	2011	Unknown	Unknown
Establishing the safety and efficacy of a new multi-agent intrathecal treatment protocol for patients with neoplastic meningitis	Zammar, S. and Eby, R. and Zacharia, B. and Strowd, R. and Grossman, S. and Aregawi, D. and Michael, G.	Zammar	2019	Retrospective study	Unknown
TBCRC 022: A Phase II Trial of Neratinib and Capecitabine for Patients With Human Epidermal Growth Factor Receptor 2–Positive Breast Cancer and Brain Metastases	Rachel A. Freedman, MD, MPH; Rebecca S. Gelman, PhD; Carey K. Anders, MD; Michelle E. Melisko, MD; Heather A. Parsons, MD1 Anne M. Cropp; Kelly Silvestri; Christine M. Cotter; Kathryn P. Componeschi, MBA; Juan M. Marte; Roisin M. Connolly, MBBCh, MD; Beverly Moy, MD; Catherine H. Van Poznak, MD; Kimberly L. Blackwell, MD; Shannon L. Puhalla, MD; Rachel C. Jankowitz, MD; Karen L. Smith, MD; Nuhad Ibrahim, MD; Timothy J. Moynihan, MD; Ciara C. O'Sullivan, MBBCh; Julie Nangia, MD; Polly Niravath, MD; Nadine Tung, MD; Paula R. Pohlmann, MD, PhD; Robyn Burns, PhD; Mothaffar F. Rimawi, MD; Ian E. Krop, MD, PhD; Antonio C. Wolff, MD; Eric P. Winer, MD; and Nancy U. Lin, MD on behalf of the Translational Breast Cancer Research Consortium	Freedman	2019	Prospective trial	3
Trastuzumab Deruxtecan in Patients with Central Nervous System Involvement from HER2-Positive Breast Cancer: The DEBBRAH Trial	José Manuel Pérez-García, Marta Vaz Batista, Patricia Cortez, Manuel Ruiz-Borrego, Juan Miguel Cejalvo, Juan de la Haba-Rodriguez, Laia Garrigós, Fabricio Racca, Sonia Servitja, Salvador Blanch, María Gion, Monica Nave, María Fernández-Abad, Alejandro Martinez-Bueno, Antonio Llombart-Cussac, Miguel Sampayo-Cordero, Andrea Malfettone, Javier Cortés, Sofía Braga	Perez Garcia	2022	Prospective trial	Unknown
					
				Total prospective	115
*Do not specify number of patients having received HER2-targeted therapy				Total retrospective	167
**Do not specify exact number of patients with LM				Total case reports/series	61
				Total	343

## Appendix 1 Detailed search strategy

### Search summary

Ovid MEDLINE(R) ALL <1946 to December 21, 2021>

1 Meningeal Carcinomatosis/674
2 ((leptomening* or leptomenix or meningeal* or meningitides) adj3 (metastas* or carcinos#s or carcinomat* or disease*)). mp. 3275
3 ((carcinomat* or neoplastic or malignan*) adj1 meningitis). mp. 691
4 ((meninges or meninx or dura or dural) adj3 carcinomat*). mp. 42
5 or/1–4 3736
6 5 not (exp animals/not humans. sh.) 3646

Embase Classic+Embase <1947 to 2021 December 21>

1 carcinomatous meningitis/2527
2 ((leptomening* or leptomenix or meningeal* or meningitides) adj3 (metastas* or carcinos#s or carcinomat* or disease*)). mp. 5689
3 ((carcinomat* or neoplastic or malignan*) adj1 meningitis). mp. 3051
4 ((meninges or meninx or dura or dural) adj3 carcinomat*). mp. 775 or/a-d 7117
6 5 not ((exp animal/or animal experiment/or nonhuman/) not (exp human/or human experiment/)) 6929

Animal-only indexed studies filter from adapted from https://www.cochranelibrary.com/central/central-creation.

Cochrane Central Register of Controlled Trials.

ID Search Hits#1 [mh ^"Meningeal Carcinomatosis"] 11 #2 ((leptomening*:ti,ab, kw OR leptomenix:ti,ab, kw OR meningeal*:ti,ab, kw OR meningitides:ti,ab,kw) NEAR/3 (metastas*:ti,ab, kw OR carcinos?s:ti,ab, kw OR carcinomat*:ti,ab, kw OR disease*:ti,ab,kw)) 128 #3 ((carcinomat*:ti,ab, kw OR neoplastic:ti,ab, kw OR malignan*:ti,ab,kw) NEAR/1 meningitis:ti,ab,kw) 48 #4 ((meninges:ti,ab, kw OR meninx:ti,ab, kw OR dura:ti,ab, kw OR dural:ti,ab,kw) NEAR/3 carcinomat*:ti,ab,kw) 0 #5 #1 OR #2 OR #3 OR #4157 Web of Science.

## Footnotes

Appendix A Supplementary data to this article can be found online at https://doi.org/10.1016/j.breast.2023.04.008.
