# Supplementary material for: Intrathecal trastuzumab versus alternate routes of delivery for HER2-targeted therapies in patients with HER2+ breast cancer leptomeningeal metastases
Source: Breast. 2023 May 1;69:451–68. doi: 10.1016/j.breast.2023.04.008 (PMC10300571; doi:10.1016/j.breast.2023.04.008)
Supplement: Multimedia component 4 [file mmc4.pptx]

## Slide 1
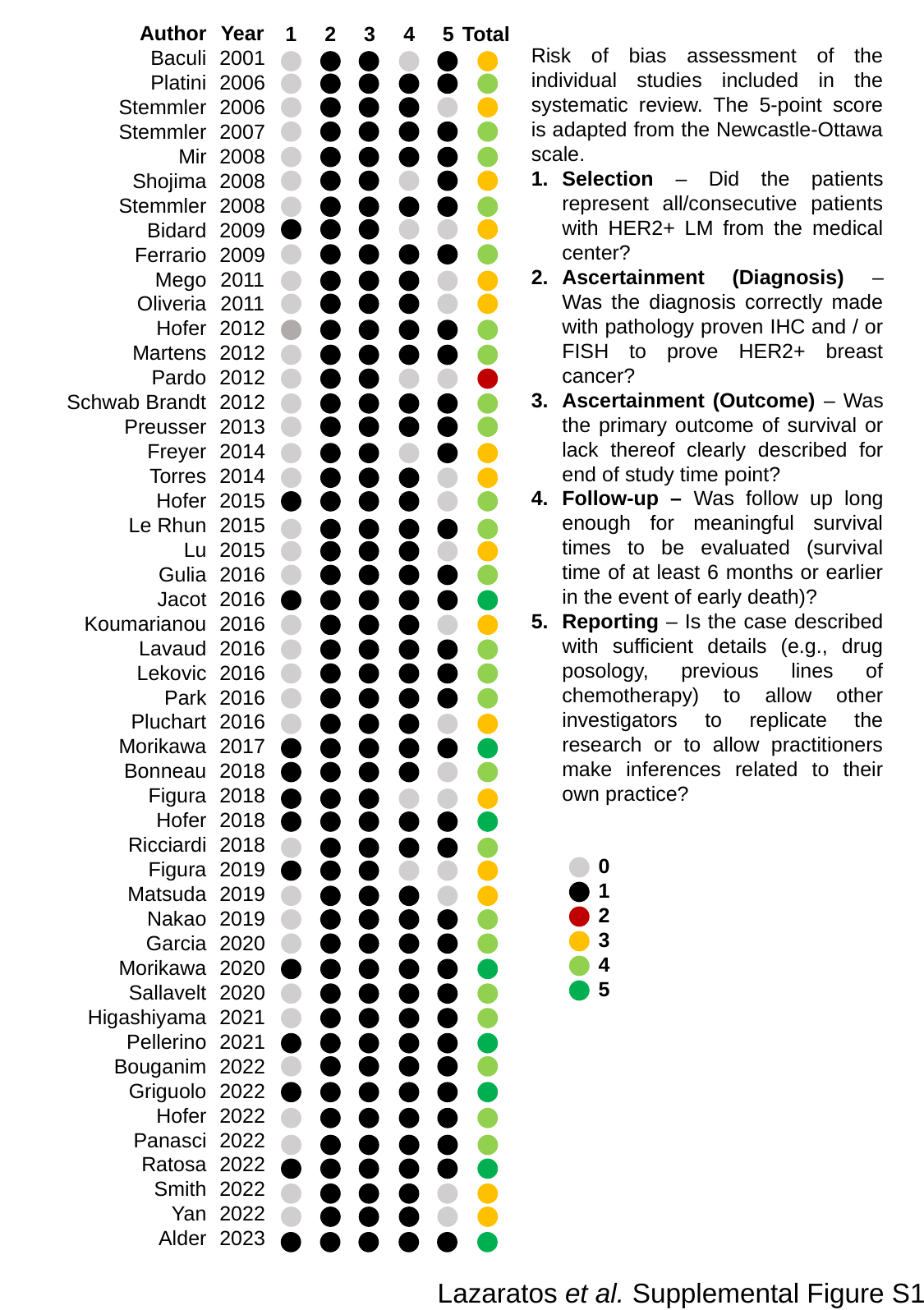

Author
Baculi
Platini
Stemmler
Stemmler
Mir
Shojima
Stemmler
Bidard
Ferrario
Mego
Oliveria
Hofer
Martens
Pardo
Schwab Brandt
Preusser
Freyer
Torres
Hofer
Le Rhun
Lu
Gulia
Jacot
Koumarianou
Lavaud
Lekovic
Park
Pluchart
Morikawa
Bonneau
Figura
Hofer
Ricciardi
Figura
Matsuda
Nakao
Garcia
Morikawa
Sallavelt
Higashiyama
Pellerino
Bouganim
Griguolo
Hofer
Panasci
Ratosa
Smith
Yan
Alder
Year
2001
2006
2006
2007
2008
2008
2008
2009
2009
2011
2011
2012
2012
2012
2012
2013
2014
2014
2015
2015
2015
2016
2016
2016
2016
2016
2016
2016
2017
2018
2018
2018
2018
2019
2019
2019
2020
2020
2020
2021
2021
2022
2022
2022
2022
2022
2022
2022
2023
Total
4
5
1
3
2
Risk of bias assessment of the individual studies included in the systematic review. The 5-point score is adapted from the Newcastle-Ottawa scale.
Selection – Did the patients represent all/consecutive patients with HER2+ LM from the medical center?
Ascertainment (Diagnosis) – Was the diagnosis correctly made with pathology proven IHC and / or FISH to prove HER2+ breast cancer?
Ascertainment (Outcome) – Was the primary outcome of survival or lack thereof clearly described for end of study time point?
Follow-up – Was follow up long enough for meaningful survival times to be evaluated (survival time of at least 6 months or earlier in the event of early death)?
Reporting – Is the case described with sufficient details (e.g., drug posology, previous lines of chemotherapy) to allow other investigators to replicate the research or to allow practitioners make inferences related to their own practice?
0
1
2
3
4
5
Lazaratos et al. Supplemental Figure S1

## Slide 2
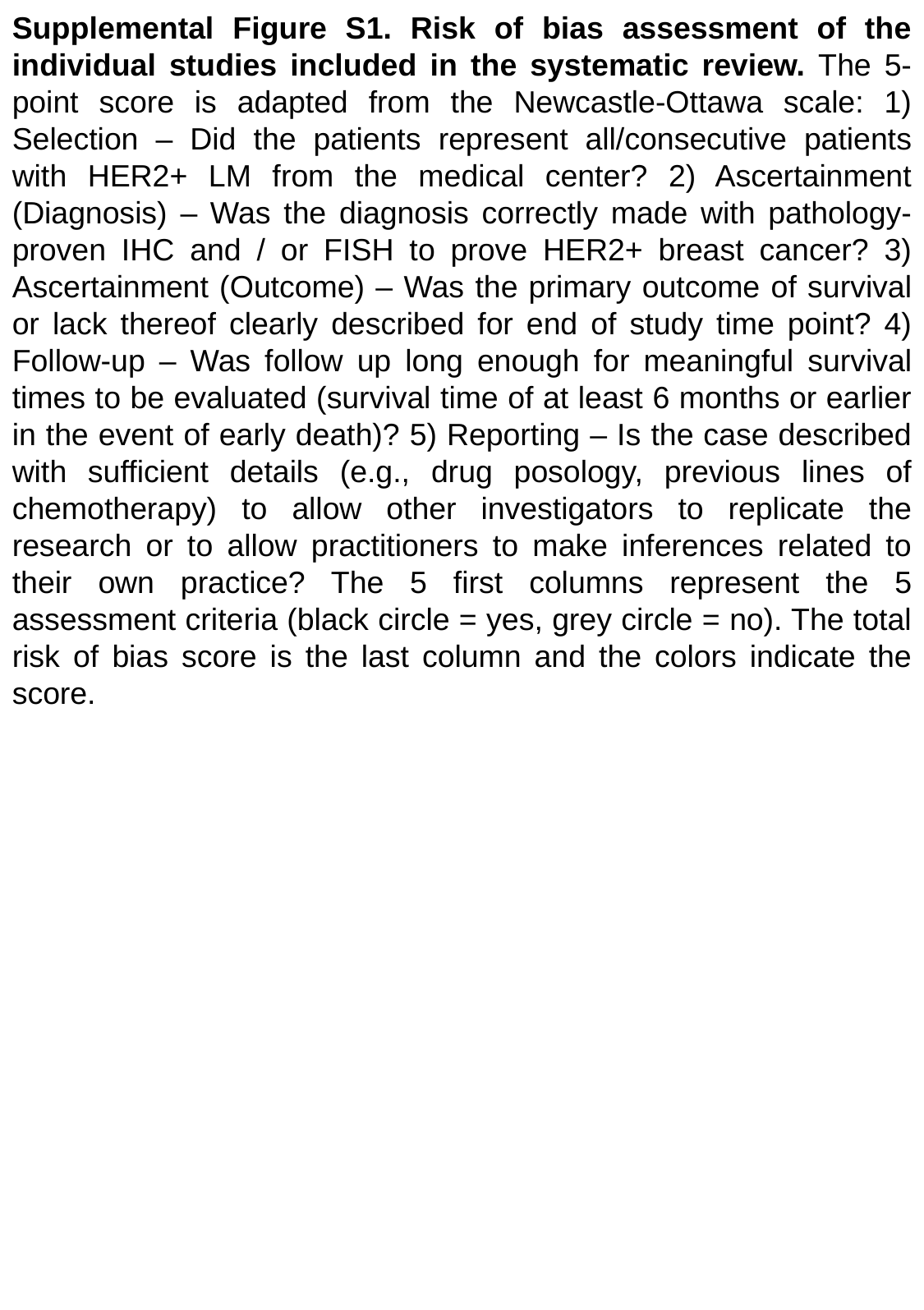

Supplemental Figure S1. Risk of bias assessment of the individual studies included in the systematic review. The 5-point score is adapted from the Newcastle-Ottawa scale: 1) Selection – Did the patients represent all/consecutive patients with HER2+ LM from the medical center? 2) Ascertainment (Diagnosis) – Was the diagnosis correctly made with pathology-proven IHC and / or FISH to prove HER2+ breast cancer? 3) Ascertainment (Outcome) – Was the primary outcome of survival or lack thereof clearly described for end of study time point? 4) Follow-up – Was follow up long enough for meaningful survival times to be evaluated (survival time of at least 6 months or earlier in the event of early death)? 5) Reporting – Is the case described with sufficient details (e.g., drug posology, previous lines of chemotherapy) to allow other investigators to replicate the research or to allow practitioners to make inferences related to their own practice? The 5 first columns represent the 5 assessment criteria (black circle = yes, grey circle = no). The total risk of bias score is the last column and the colors indicate the score.
