# Supplementary material for: Intrathecal trastuzumab versus alternate routes of delivery for HER2-targeted therapies in patients with HER2+ breast cancer leptomeningeal metastases
Source: Breast. 2023 May 1;69:451–68. doi: 10.1016/j.breast.2023.04.008 (PMC10300571; doi:10.1016/j.breast.2023.04.008)
Supplement: Multimedia component 5 [file mmc5.pptx]

## Slide 1
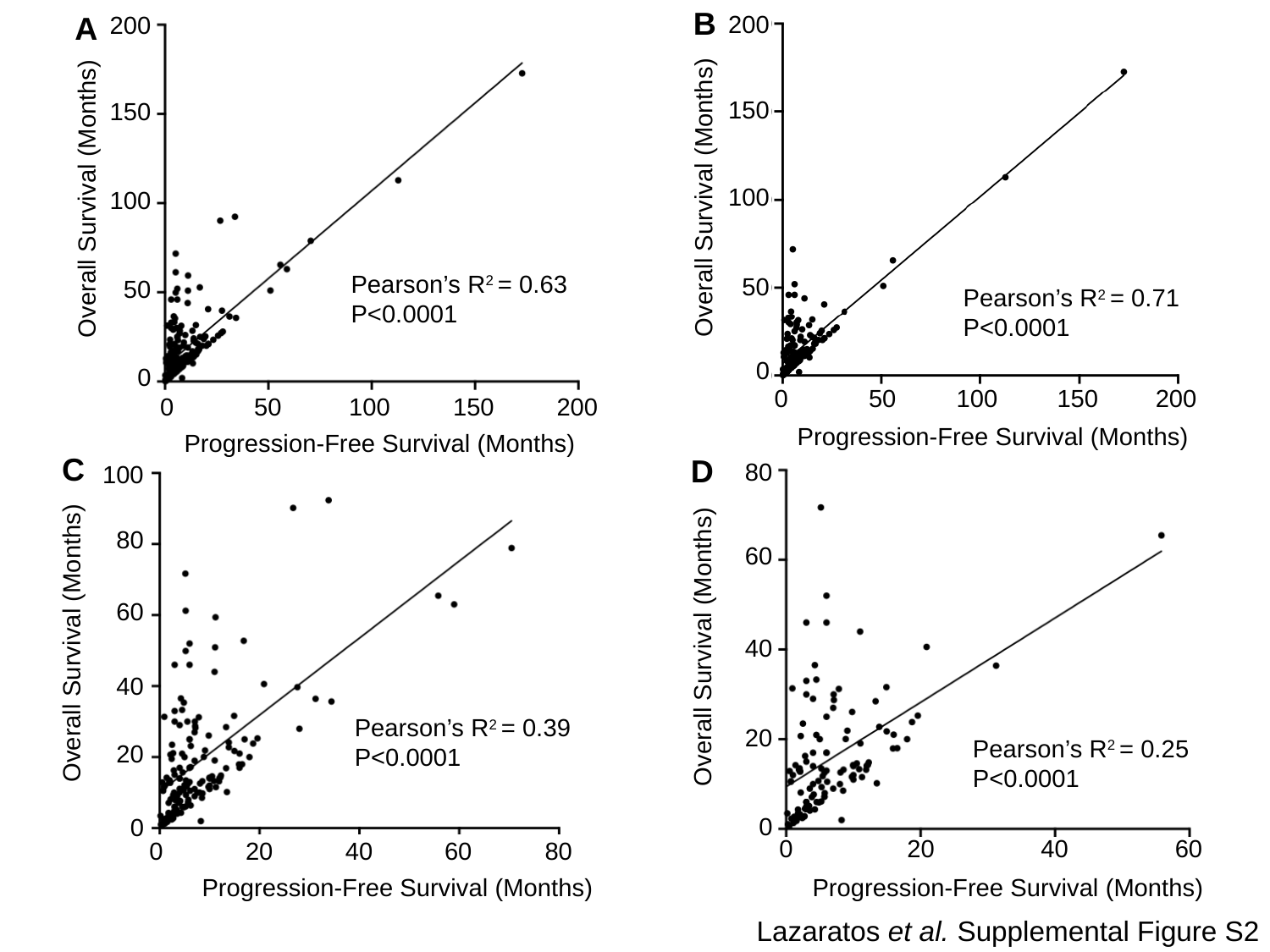

B
200
150
100
Overall Survival (Months)
50
Pearson’s R2 = 0.71
P<0.0001
 0
 0
50
100
150
200
Progression-Free Survival (Months)
A
200
150
Overall Survival (Months)
100
Pearson’s R2 = 0.63
P<0.0001
50
 0
 0
50
100
150
200
Progression-Free Survival (Months)
C
100
80
60
Overall Survival (Months)
40
Pearson’s R2 = 0.39
P<0.0001
20
 0
 0
20
40
60
80
Progression-Free Survival (Months)
D
80
60
Overall Survival (Months)
40
20
Pearson’s R2 = 0.25
P<0.0001
 0
 0
20
40
60
Progression-Free Survival (Months)
Lazaratos et al. Supplemental Figure S2

## Slide 2
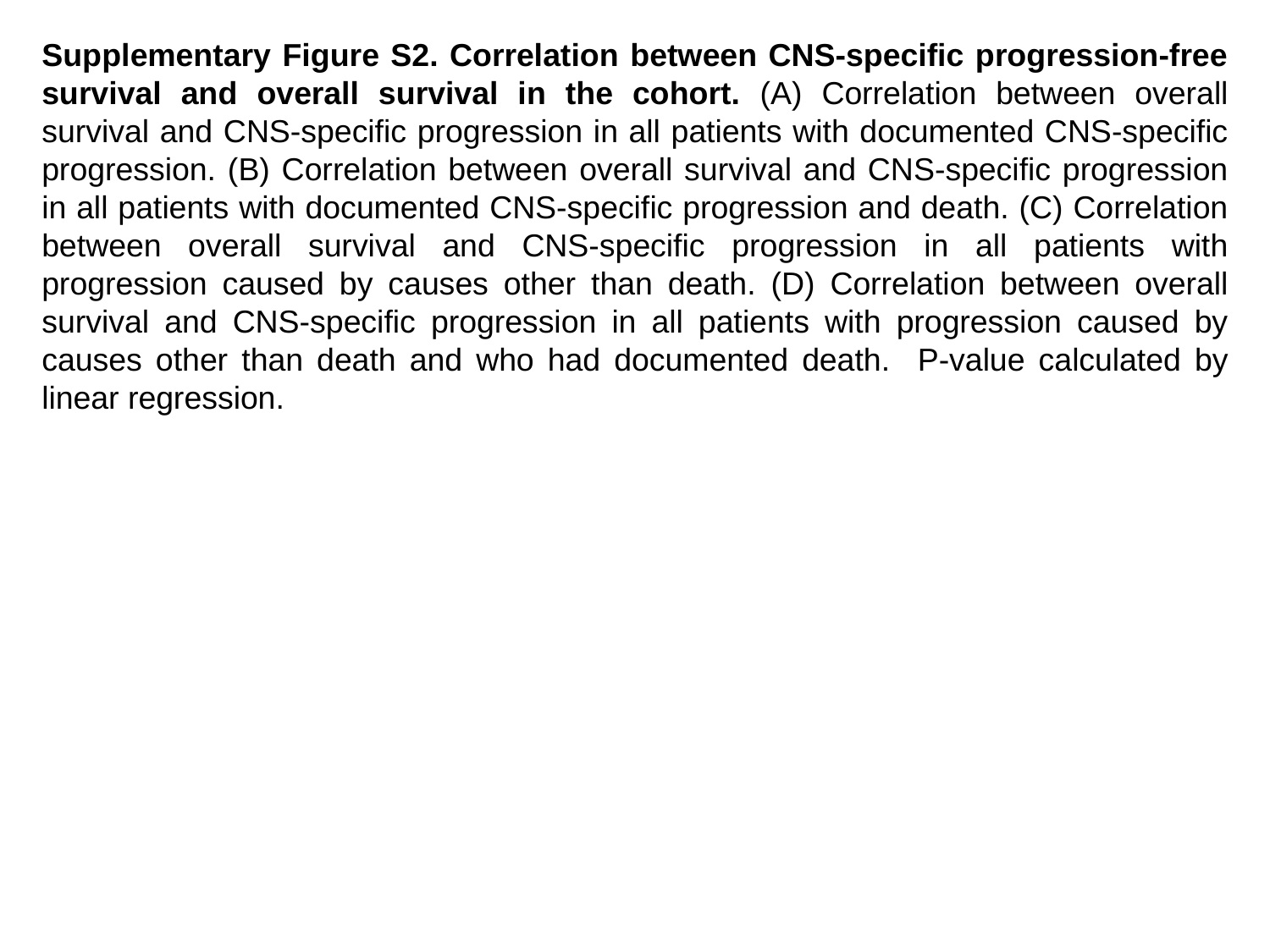

Supplementary Figure S2. Correlation between CNS-specific progression-free survival and overall survival in the cohort. (A) Correlation between overall survival and CNS-specific progression in all patients with documented CNS-specific progression. (B) Correlation between overall survival and CNS-specific progression in all patients with documented CNS-specific progression and death. (C) Correlation between overall survival and CNS-specific progression in all patients with progression caused by causes other than death. (D) Correlation between overall survival and CNS-specific progression in all patients with progression caused by causes other than death and who had documented death. P-value calculated by linear regression.
