# Supplementary material for: Intrathecal trastuzumab versus alternate routes of delivery for HER2-targeted therapies in patients with HER2+ breast cancer leptomeningeal metastases
Source: Breast. 2023 May 1;69:451–68. doi: 10.1016/j.breast.2023.04.008 (PMC10300571; doi:10.1016/j.breast.2023.04.008)
Supplement: Multimedia component 6 [file mmc6.pptx]

## Slide 1
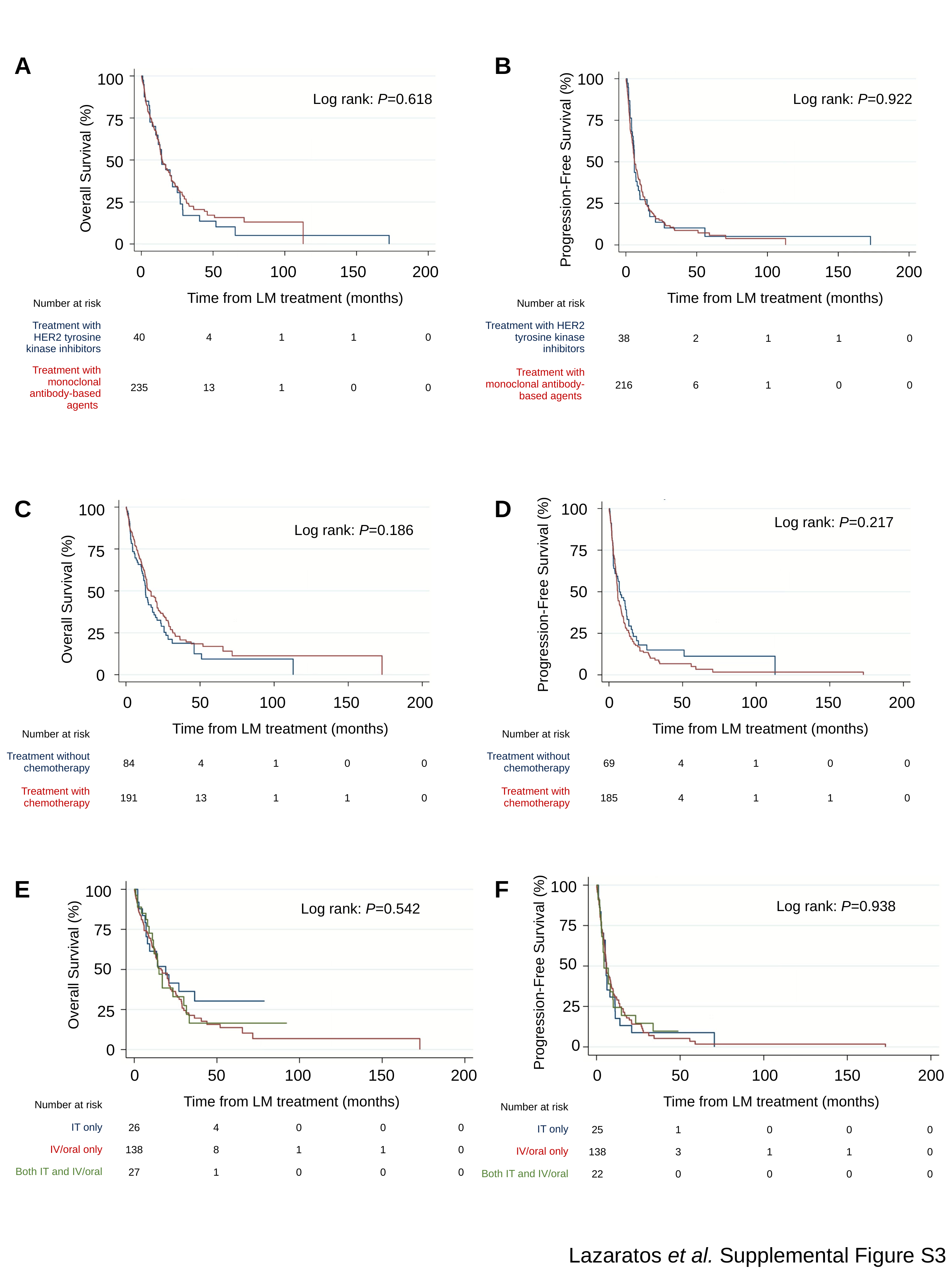

A
B
100
100
Log rank: P=0.618
Log rank: P=0.922
75
75
50
50
Overall Survival (%)
Progression-Free Survival (%)
25
25
0
0
0
50
100
150
200
0
50
100
150
200
Time from LM treatment (months)
Time from LM treatment (months)
| Number at risk | | | | | |
| --- | --- | --- | --- | --- | --- |
| Treatment with HER2 tyrosine kinase inhibitors | 40 | 4 | 1 | 1 | 0 |
| Treatment with monoclonal antibody-based agents | 235 | 13 | 1 | 0 | 0 |
| Number at risk | | | | | |
| --- | --- | --- | --- | --- | --- |
| Treatment with HER2 tyrosine kinase inhibitors | 38 | 2 | 1 | 1 | 0 |
| Treatment with monoclonal antibody-based agents | 216 | 6 | 1 | 0 | 0 |
C
D
100
100
Log rank: P=0.217
Log rank: P=0.186
75
75
50
50
Progression-Free Survival (%)
Overall Survival (%)
25
25
0
0
0
50
100
150
200
0
50
100
150
200
Time from LM treatment (months)
Time from LM treatment (months)
| Number at risk | | | | | |
| --- | --- | --- | --- | --- | --- |
| Treatment without chemotherapy | 84 | 4 | 1 | 0 | 0 |
| Treatment with chemotherapy | 191 | 13 | 1 | 1 | 0 |
| Number at risk | | | | | |
| --- | --- | --- | --- | --- | --- |
| Treatment without chemotherapy | 69 | 4 | 1 | 0 | 0 |
| Treatment with chemotherapy | 185 | 4 | 1 | 1 | 0 |
E
F
100
100
Log rank: P=0.938
Log rank: P=0.542
75
75
50
Overall Survival (%)
50
Progression-Free Survival (%)
25
25
0
0
0
50
100
150
200
0
50
100
150
200
Time from LM treatment (months)
Time from LM treatment (months)
| Number at risk | | | | | |
| --- | --- | --- | --- | --- | --- |
| IT only | 26 | 4 | 0 | 0 | 0 |
| IV/oral only | 138 | 8 | 1 | 1 | 0 |
| Both IT and IV/oral | 27 | 1 | 0 | 0 | 0 |
| Number at risk | | | | | |
| --- | --- | --- | --- | --- | --- |
| IT only | 25 | 1 | 0 | 0 | 0 |
| IV/oral only | 138 | 3 | 1 | 1 | 0 |
| Both IT and IV/oral | 22 | 0 | 0 | 0 | 0 |
Lazaratos et al. Supplemental Figure S3

## Slide 2
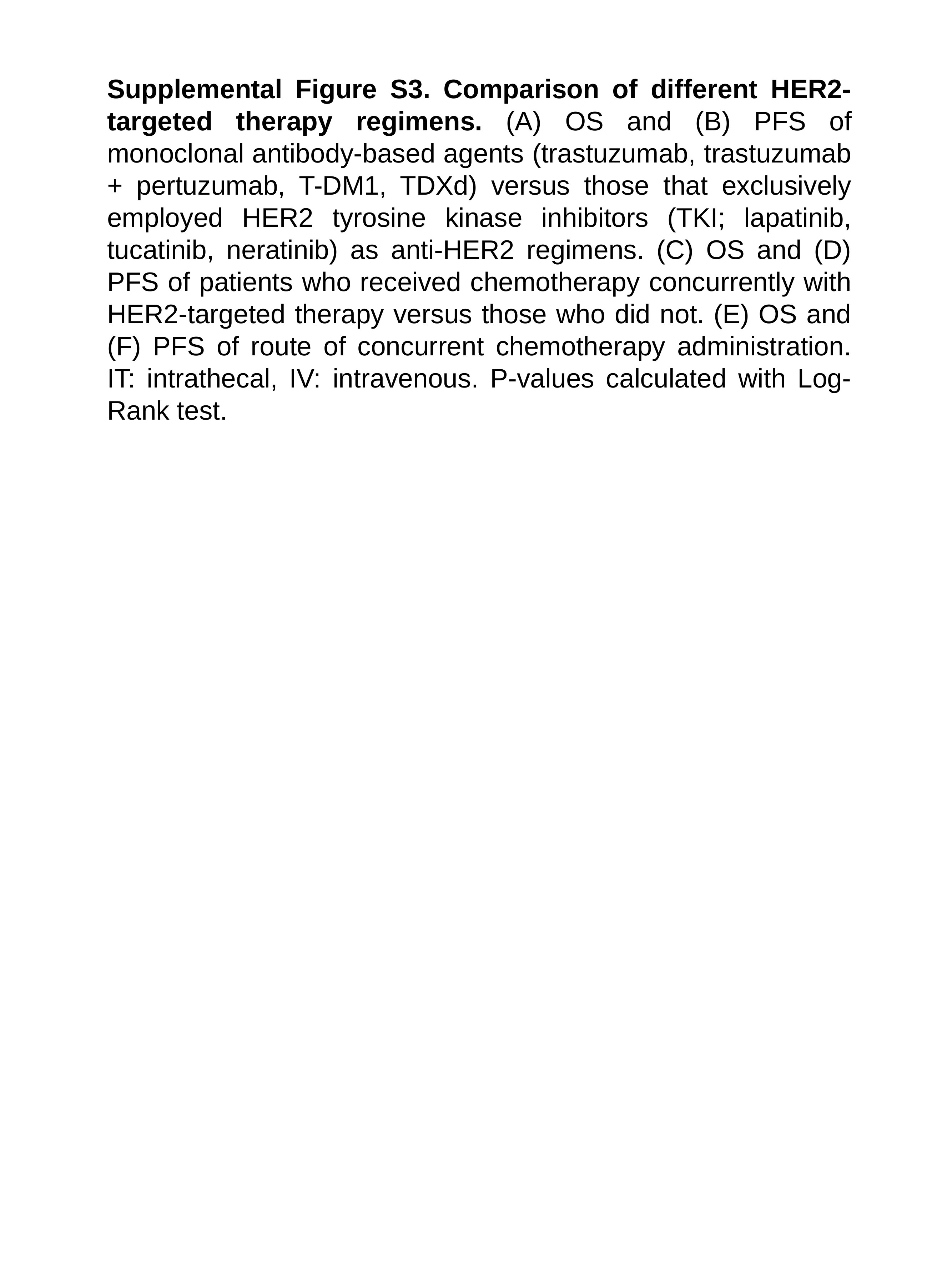

Supplemental Figure S3. Comparison of different HER2-targeted therapy regimens. (A) OS and (B) PFS of monoclonal antibody-based agents (trastuzumab, trastuzumab + pertuzumab, T-DM1, TDXd) versus those that exclusively employed HER2 tyrosine kinase inhibitors (TKI; lapatinib, tucatinib, neratinib) as anti-HER2 regimens. (C) OS and (D) PFS of patients who received chemotherapy concurrently with HER2-targeted therapy versus those who did not. (E) OS and (F) PFS of route of concurrent chemotherapy administration. IT: intrathecal, IV: intravenous. P-values calculated with Log-Rank test.
