# Supplementary material for: Intrathecal trastuzumab versus alternate routes of delivery for HER2-targeted therapies in patients with HER2+ breast cancer leptomeningeal metastases
Source: Breast. 2023 May 1;69:451–68. doi: 10.1016/j.breast.2023.04.008 (PMC10300571; doi:10.1016/j.breast.2023.04.008)
Supplement: Multimedia component 7 [file mmc7.pptx]

## Slide 1
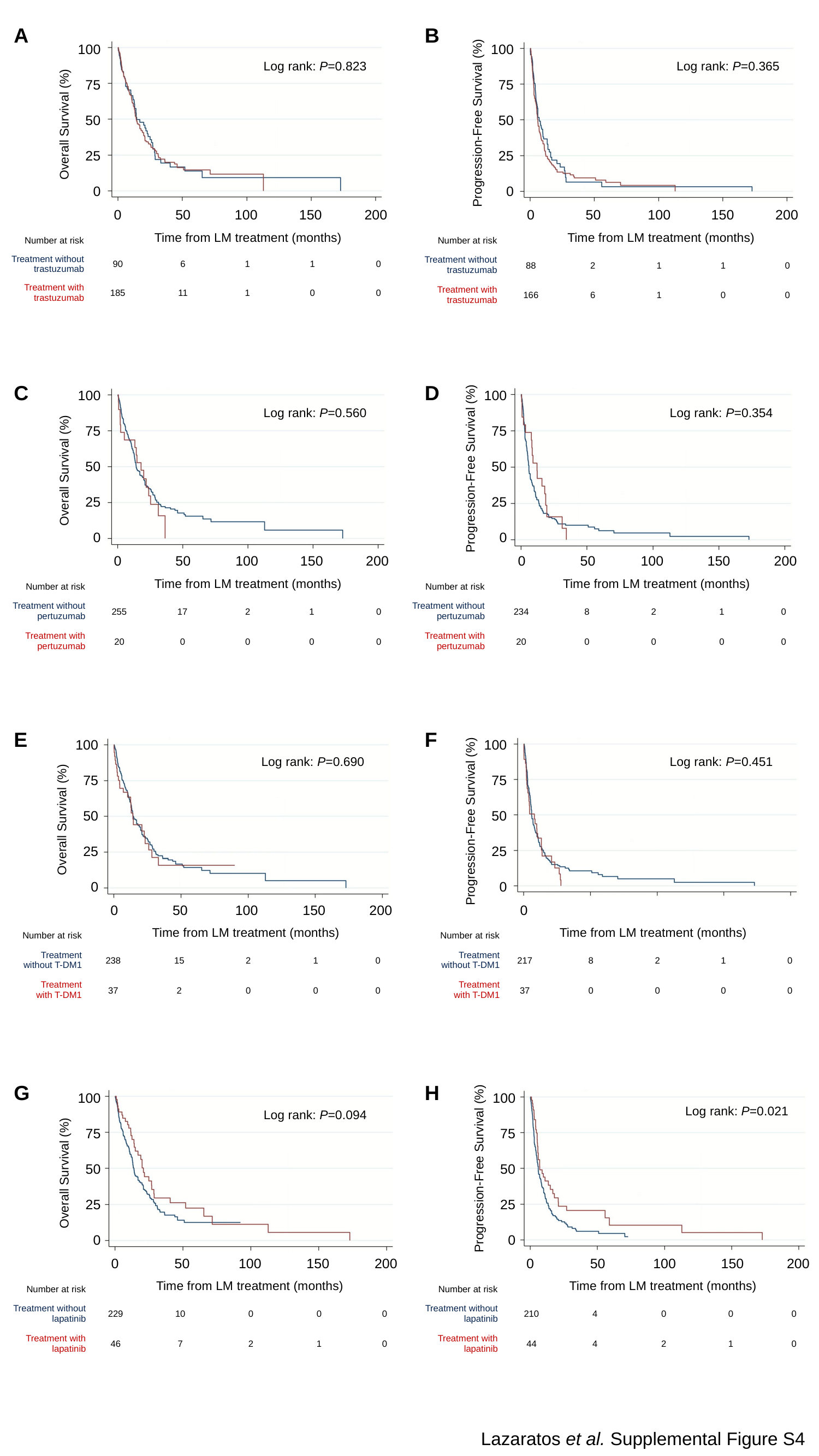

A
B
100
100
Log rank: P=0.823
Log rank: P=0.365
75
75
50
50
Progression-Free Survival (%)
Overall Survival (%)
25
25
0
0
0
50
100
150
200
0
50
100
150
200
Time from LM treatment (months)
Time from LM treatment (months)
| Number at risk | | | | | |
| --- | --- | --- | --- | --- | --- |
| Treatment without trastuzumab | 90 | 6 | 1 | 1 | 0 |
| Treatment with trastuzumab | 185 | 11 | 1 | 0 | 0 |
| Number at risk | | | | | |
| --- | --- | --- | --- | --- | --- |
| Treatment without trastuzumab | 88 | 2 | 1 | 1 | 0 |
| Treatment with trastuzumab | 166 | 6 | 1 | 0 | 0 |
C
D
100
100
Log rank: P=0.560
Log rank: P=0.354
75
75
50
50
Progression-Free Survival (%)
Overall Survival (%)
25
25
0
0
0
50
100
150
200
0
50
100
150
200
Time from LM treatment (months)
Time from LM treatment (months)
| Number at risk | | | | | |
| --- | --- | --- | --- | --- | --- |
| Treatment without pertuzumab | 255 | 17 | 2 | 1 | 0 |
| Treatment with pertuzumab | 20 | 0 | 0 | 0 | 0 |
| Number at risk | | | | | |
| --- | --- | --- | --- | --- | --- |
| Treatment without pertuzumab | 234 | 8 | 2 | 1 | 0 |
| Treatment with pertuzumab | 20 | 0 | 0 | 0 | 0 |
E
F
100
100
Log rank: P=0.690
Log rank: P=0.451
75
75
50
50
Overall Survival (%)
Progression-Free Survival (%)
25
25
0
0
0
50
100
150
200
0
Time from LM treatment (months)
Time from LM treatment (months)
| Number at risk | | | | | |
| --- | --- | --- | --- | --- | --- |
| Treatment without T-DM1 | 238 | 15 | 2 | 1 | 0 |
| Treatment with T-DM1 | 37 | 2 | 0 | 0 | 0 |
| Number at risk | | | | | |
| --- | --- | --- | --- | --- | --- |
| Treatment without T-DM1 | 217 | 8 | 2 | 1 | 0 |
| Treatment with T-DM1 | 37 | 0 | 0 | 0 | 0 |
G
H
100
100
Log rank: P=0.021
Log rank: P=0.094
75
75
50
50
Progression-Free Survival (%)
Overall Survival (%)
25
25
0
0
0
50
100
150
200
0
50
100
150
200
Time from LM treatment (months)
Time from LM treatment (months)
| Number at risk | | | | | |
| --- | --- | --- | --- | --- | --- |
| Treatment without lapatinib | 229 | 10 | 0 | 0 | 0 |
| Treatment with lapatinib | 46 | 7 | 2 | 1 | 0 |
| Number at risk | | | | | |
| --- | --- | --- | --- | --- | --- |
| Treatment without lapatinib | 210 | 4 | 0 | 0 | 0 |
| Treatment with lapatinib | 44 | 4 | 2 | 1 | 0 |
Lazaratos et al. Supplemental Figure S4

## Slide 2
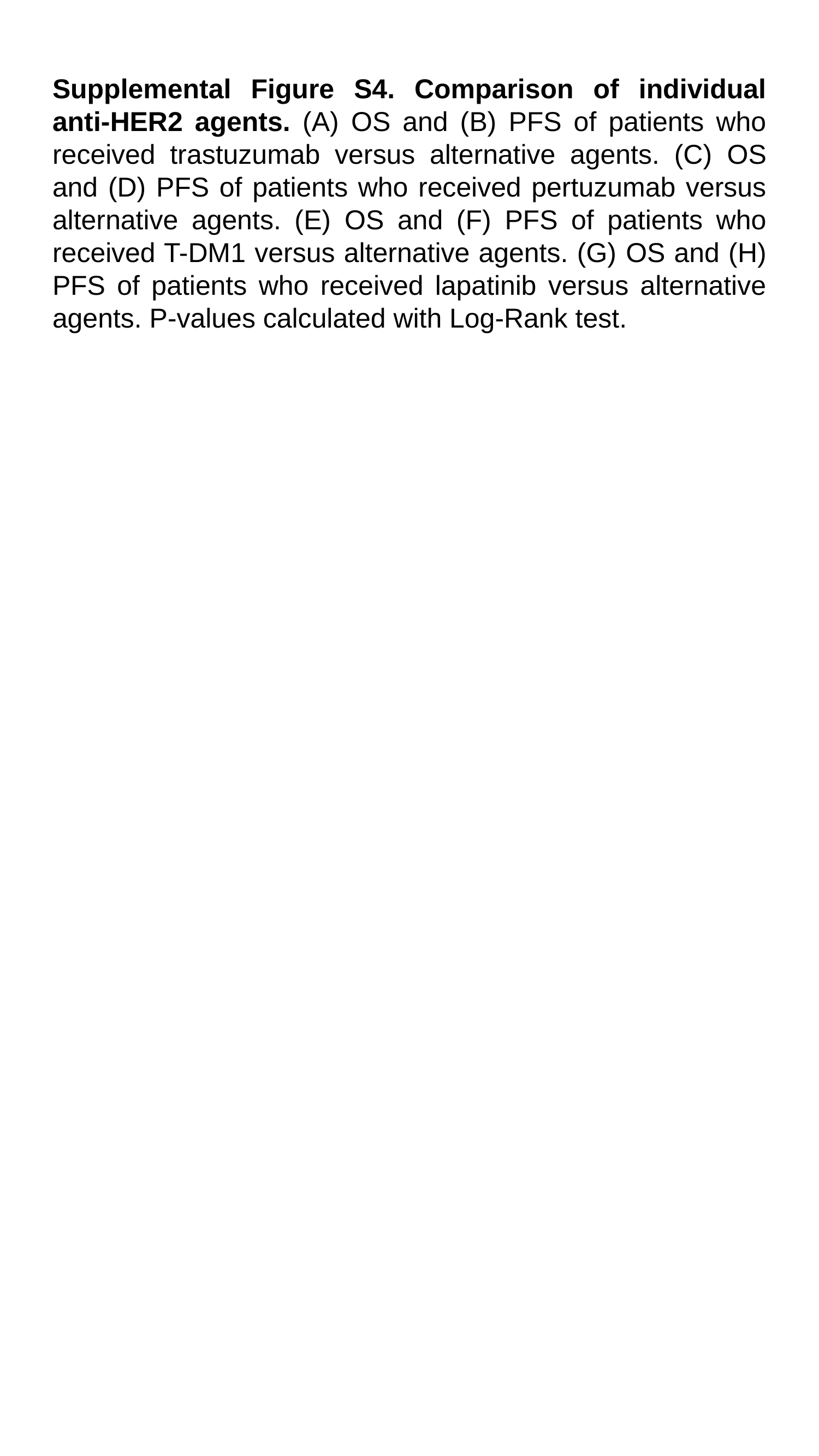

Supplemental Figure S4. Comparison of individual anti-HER2 agents. (A) OS and (B) PFS of patients who received trastuzumab versus alternative agents. (C) OS and (D) PFS of patients who received pertuzumab versus alternative agents. (E) OS and (F) PFS of patients who received T-DM1 versus alternative agents. (G) OS and (H) PFS of patients who received lapatinib versus alternative agents. P-values calculated with Log-Rank test.
