# Supplementary material for: Intrathecal trastuzumab versus alternate routes of delivery for HER2-targeted therapies in patients with HER2+ breast cancer leptomeningeal metastases
Source: Breast. 2023 May 1;69:451–68. doi: 10.1016/j.breast.2023.04.008 (PMC10300571; doi:10.1016/j.breast.2023.04.008)
Supplement: Multimedia component 8 [file mmc8.pptx]

## Slide 1
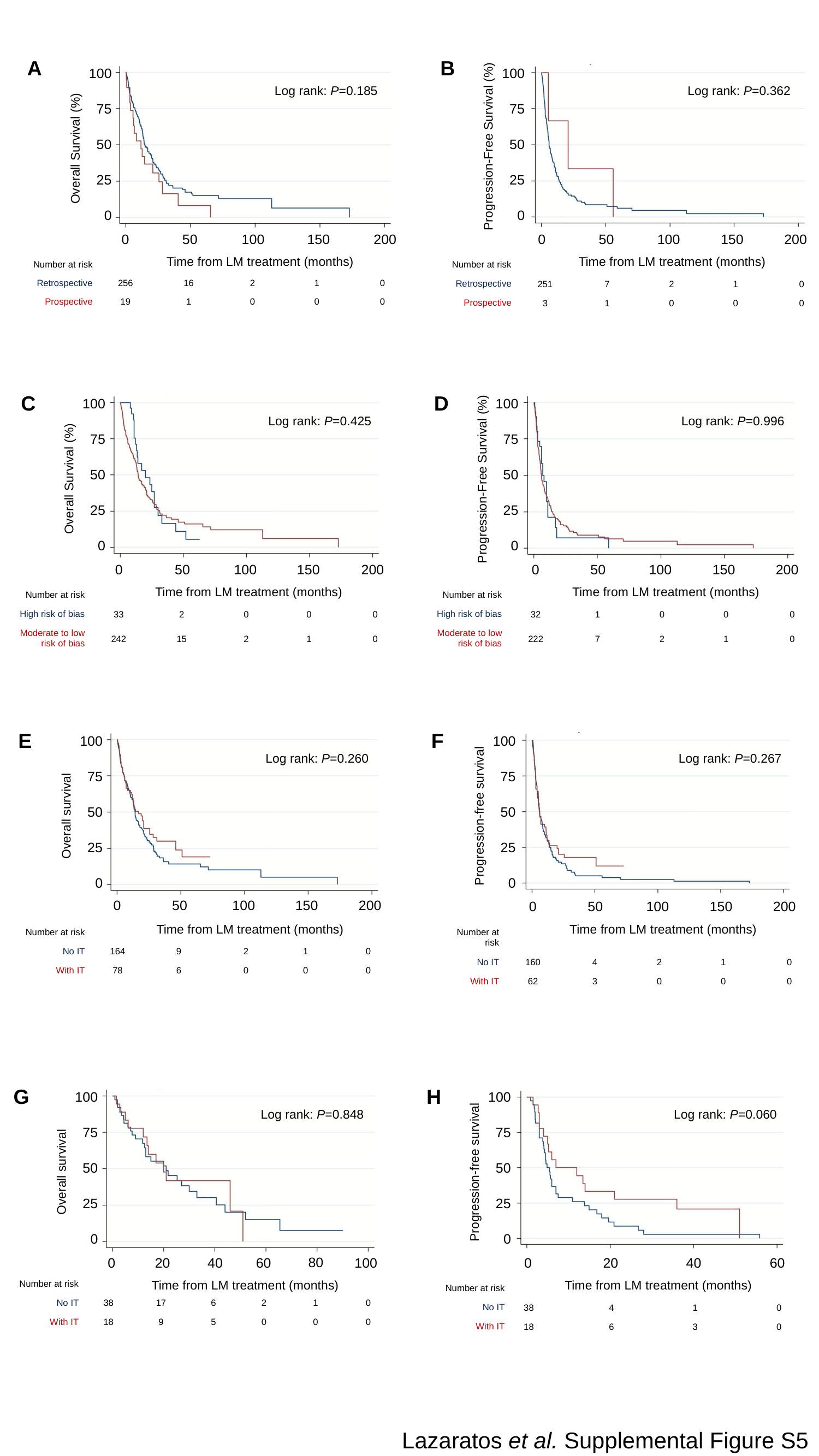

A
B
100
100
Log rank: P=0.185
Log rank: P=0.362
75
75
50
50
Progression-Free Survival (%)
Overall Survival (%)
25
25
0
0
0
50
100
150
200
0
50
100
150
200
Time from LM treatment (months)
Time from LM treatment (months)
| Number at risk | | | | | |
| --- | --- | --- | --- | --- | --- |
| Retrospective | 256 | 16 | 2 | 1 | 0 |
| Prospective | 19 | 1 | 0 | 0 | 0 |
| Number at risk | | | | | |
| --- | --- | --- | --- | --- | --- |
| Retrospective | 251 | 7 | 2 | 1 | 0 |
| Prospective | 3 | 1 | 0 | 0 | 0 |
C
D
100
100
Log rank: P=0.425
Log rank: P=0.996
75
75
50
50
Overall Survival (%)
Progression-Free Survival (%)
25
25
0
0
0
50
100
150
200
0
50
100
150
200
Time from LM treatment (months)
Time from LM treatment (months)
| Number at risk | | | | | |
| --- | --- | --- | --- | --- | --- |
| High risk of bias | 33 | 2 | 0 | 0 | 0 |
| Moderate to low risk of bias | 242 | 15 | 2 | 1 | 0 |
| Number at risk | | | | | |
| --- | --- | --- | --- | --- | --- |
| High risk of bias | 32 | 1 | 0 | 0 | 0 |
| Moderate to low risk of bias | 222 | 7 | 2 | 1 | 0 |
E
F
100
100
Log rank: P=0.260
Log rank: P=0.267
75
75
50
50
Overall survival
Progression-free survival
25
25
0
0
0
50
100
150
200
0
50
100
150
200
Time from LM treatment (months)
Time from LM treatment (months)
| Number at risk | | | | | |
| --- | --- | --- | --- | --- | --- |
| No IT | 164 | 9 | 2 | 1 | 0 |
| With IT | 78 | 6 | 0 | 0 | 0 |
| Number at risk | | | | | |
| --- | --- | --- | --- | --- | --- |
| No IT | 160 | 4 | 2 | 1 | 0 |
| With IT | 62 | 3 | 0 | 0 | 0 |
G
H
100
100
Log rank: P=0.848
Log rank: P=0.060
75
75
50
50
Overall survival
Progression-free survival
25
25
0
0
80
0
20
40
60
100
0
20
40
60
Time from LM treatment (months)
Time from LM treatment (months)
| Number at risk | | | | | | |
| --- | --- | --- | --- | --- | --- | --- |
| No IT | 38 | 17 | 6 | 2 | 1 | 0 |
| With IT | 18 | 9 | 5 | 0 | 0 | 0 |
| Number at risk | | | | |
| --- | --- | --- | --- | --- |
| No IT | 38 | 4 | 1 | 0 |
| With IT | 18 | 6 | 3 | 0 |
Lazaratos et al. Supplemental Figure S5

## Slide 2
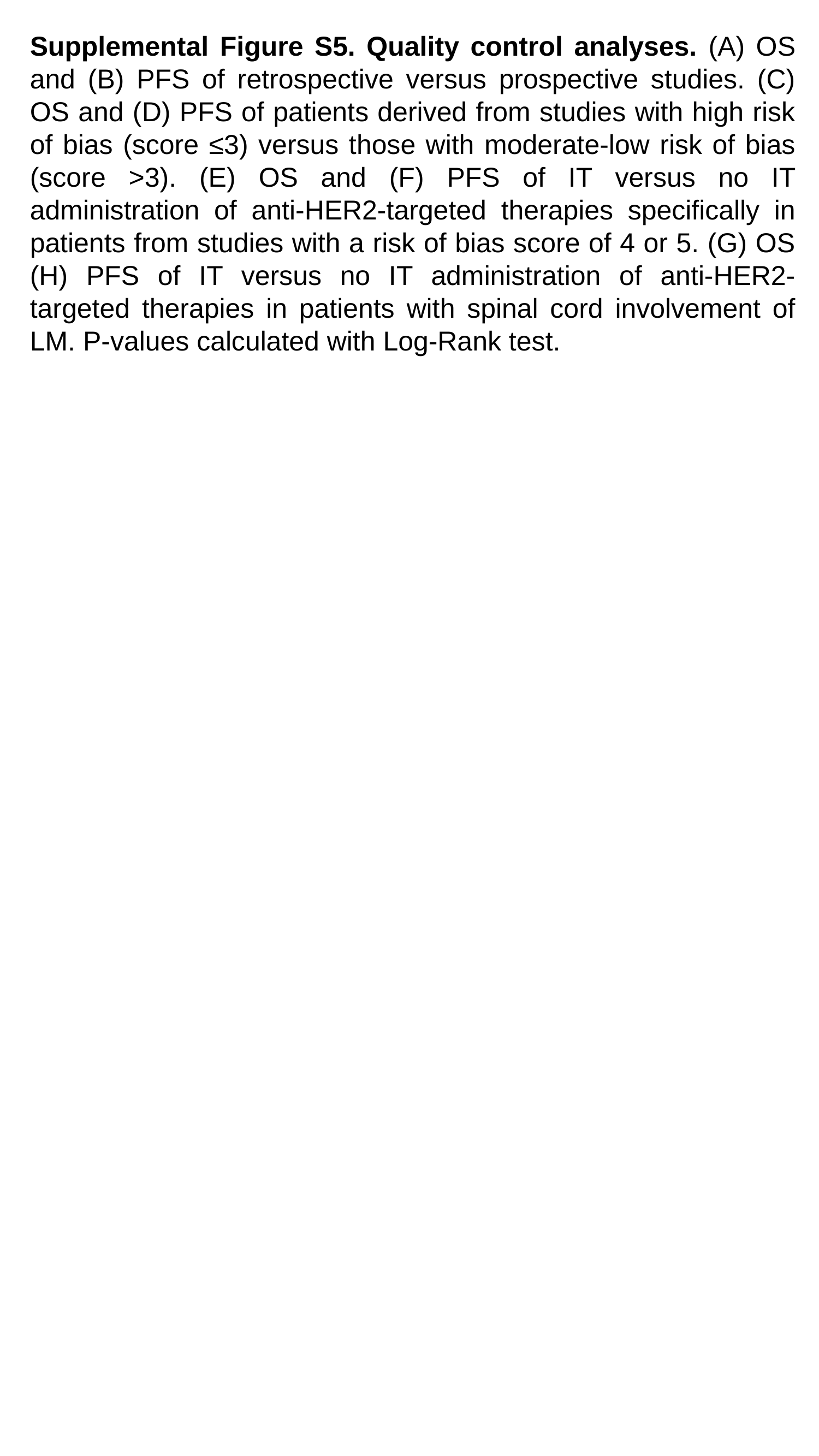

Supplemental Figure S5. Quality control analyses. (A) OS and (B) PFS of retrospective versus prospective studies. (C) OS and (D) PFS of patients derived from studies with high risk of bias (score ≤3) versus those with moderate-low risk of bias (score >3). (E) OS and (F) PFS of IT versus no IT administration of anti-HER2-targeted therapies specifically in patients from studies with a risk of bias score of 4 or 5. (G) OS (H) PFS of IT versus no IT administration of anti-HER2-targeted therapies in patients with spinal cord involvement of LM. P-values calculated with Log-Rank test.
